# False Morphology of Aerogels Caused by Gold Coating for SEM Imaging

**DOI:** 10.3390/polym13040588

**Published:** 2021-02-16

**Authors:** Laura Juhász, Krisztián Moldován, Pavel Gurikov, Falk Liebner, István Fábián, József Kalmár, Csaba Cserháti

**Affiliations:** 1 Department of Solid State Physics, University of Debrecen, Egyetem sqr. 1, H-4032 Debrecen, Hungary; juhasz.laura@science.unideb.hu; 2 Doctoral School of Physics, University of Debrecen, Egyetem sqr. 1, H-4032 Debrecen, Hungary; 3 MTA-DE Redox and Homogeneous Catalytic Reaction Mechanisms Research Group, Department of Inorganic and Analytical Chemistry, University of Debrecen, Egyetem sqr. 1, H-4032 Debrecen, Hungary; moldovan.krisztian@science.unideb.hu (K.M.); ifabian@science.unideb.hu (I.F.); 4 Doctoral School of Chemistry, University of Debrecen, Egyetem sqr. 1, H-4032 Debrecen, Hungary; 5 Laboratory for Development and Modelling of Novel Nanoporous Materials, Hamburg University of Technology, Eißendorfer Straße 38, 21073 Hamburg, Germany; pavel.gurikov@tuhh.de; 6 Institute for Chemistry of Renewable Resources, University of Natural Resources and Life Sciences, Vienna (BOKU), Konrad-Lorenz-Straße 24, A-3430 Tulln, Austria; falk.liebner@boku.ac.at

**Keywords:** mesoporous materials, aerogels, electron microscopy, gold sputtering, aggregation

## Abstract

The imaging of non-conducting materials by scanning electron microscopy (SEM) is most often performed after depositing few nanometers thick conductive layers on the samples. It is shown in this work, that even a 5 nm thick sputtered gold layer can dramatically alter the morphology and the surface structure of many different types of aerogels. Silica, polyimide, polyamide, calcium-alginate and cellulose aerogels were imaged in their pristine forms and after gold sputtering utilizing low voltage scanning electron microscopy (LVSEM) in order to reduce charging effects. The morphological features seen in the SEM images of the pristine samples are in excellent agreement with the structural parameters of the aerogels measured by nitrogen adsorption-desorption porosimetry. In contrast, the morphologies of the sputter coated samples are significantly distorted and feature nanostructured gold. These findings point out that extra care should be taken in order to ensure that gold sputtering does not cause morphological artifacts. Otherwise, the application of low voltage scanning electron microscopy even yields high resolution images of pristine non-conducting aerogels.

## 1. Introduction

Aerogels obtained by sol-gel and dissolution-coagulation techniques and final supercritical drying are solid functional materials of extremely high porosities and low densities. These properties are utilized in a wide range of applications including advanced thermal insulation, catalysis, manufacture of electrode materials, high-capacity adsorbents, drug delivery and tissue engineering [1,2]. Functional aerogels are prepared from a large variety of structural materials, such as inorganic oxides (e.g., silica, alumina, titania), carbohydrate polymers (e.g., cellulose, alginate, starch, chitosan, pectin), proteins (e.g., collagen, casein, egg yolk), synthetic polymers (e.g., polyimide, polyamide, polyurea) or carbon (e.g., amorphous carbon, graphene) [3,4,5,6,7,8,9,10,11,12].

In general, aerogels obtained by supercritical drying feature the best preserved three-dimensional structure of the precursor gel among all drying approaches. Hence, structural elements, including variations in the geometry of the network forming entities down to the nanoscale, can be differentiated. These differences in fine structure are related to the chemical composition of the source material(s) and the conditions of gel syntheses [13,14,15,16]. The archetypes of aerogels (silica, cellulose, alginate, polyurea, etc.) are either composed of small globular primary units of a few to a few tens of nanometers in diameter, or cylindrical fibrils of thicknesses in approximately the same size range [17,18,19]. However, some carbon based and polymer aerogels consist of flake-like flat nanostructures [20,21,22]. The pore systems of the aerogels in terms of void fraction, geometry, size distribution and degree of interconnectivity is predetermined by the architectures of their solid backbones [14,23]. The pore sizes vary between the micropore and the mesopore range, and the shapes of the pores can be spherical, cylindrical to slit-like.

The fundamental macroscopic properties (heat conductivity, compressive strength, bulk density, accessible specific surface area, total pore volume, permeability of pores, etc.) that determine the performances of the aerogels in practical applications are directly related to their microstructures [24,25,26,27]. Evidently, understanding functionality related structure-properties relationships are of key importance for designing new materials for specific advanced applications [28,29,30,31]. Furthermore, the understanding of the microstructures of aerogels enables high level theoretical simulations of their macroscopic properties [32,33].

Because of these reasons, significant efforts have been made to thoroughly characterize the nanostructured interior of these sensitive materials. Imaging the morphology by scanning electron microscopy (SEM), and assessment of pore characteristics by nitrogen adsorption-desorption porosimetry are key analytical procedures [34,35,36]. These techniques, however, require extensive care to ensure that the results are representative for the true interior of the materials, and exclude any possible artifacts. Erroneous conclusions with regard to nanostructures and morphologies of aerogels would jeopardize or even mislead time-consuming simulation efforts, and evidently, falsify structure-property relationship theories.

In this study, we systematically evaluate the performance of SEM applied to image the nanostructures of several different types of non-conducting aerogels (silica, silica hybrids, calcium-alginate, polyimide, polyamide, cellulose). The investigation of non-conducting materials commonly requires the deposition of a few nanometers thick conductive coating on the surfaces of the nanostructured samples. Here, we present clear evidence that the sputtering of gold onto the surfaces of non-conducting aerogels can significantly alter their nanostructures, thus leading to erroneous conclusions with regard to their morphologies. The imaging of pristine (uncoated) aerogel samples is also performed by using a special microscopy setup, and the observed morphological features are correlated to structural parameters derived from N${}_{2}$ adsorption-desorption porosimetry measurements [37].

Aerogel samples were characterized by Low Voltage Scanning Electron Microscopy (LVSEM). The advantage of using LVSEM has been discussed since the end of 1980s [38]. Currently, even entry-level instruments can image at low kV (1–5 kV) without much effort. This was not the case earlier. In addition, in the past, SEM instruments had significantly lower imaging resolution at low kV. The solution to this—besides the Field Emission Gun (FEG) source—are unique electron optical elements, as well as the annular in-column detector system developed by Zeiss with the GEMINI column at the beginning of 1990. This provides low kV with a very high probe current and maintains a very small probe diameter that gives a very high resolution with great signal to noise ratio and minimal sample damage. Today almost all SEM manufacturers apply these technical innovations in order to expand the capabilities of their instruments. In the present paper, we implement the newest results and innovations that are available on the market for a mainstream user.

## 2. Experimental

### 2.1. Preparation of Aerogels

Silica, silica-casein hybrid, silica-gelatin hybrid, Ca-alginate, polyimide, polyamide, Ca(II) crosslinked polyamide and cellulose aerogels were prepared using recipes previously published in the literature [20,21,22,39,40,41,42,43,44,45]. The essential summaries of the synthetic procedures are given in the Supplementary Materials.

### 2.2. Characterization of Aerogels

The main goal of the experiments was to investigate the effect of gold sputtering on the morphologies of different aerogels. The investigation was performed in four steps. First, each aerogel was imaged by SEM in its pristine form without gold coating. Second, a ca. 5 nm thick gold layer was deposited on the surfaces of the aerogels by using a conventional sputtering instrument (BIO-RAD SEM Coating Unit PS3, BIO-RAD Laboratories Ltd., Hercules, CA, USA), and the sample was imaged again. The third and fourth steps were repeating the deposition procedure in order to obtain thick, 16 nm and subsequently 32 nm coating layers before imaging. Gold deposition was carried out in 21 Pa of Ar and the sputtering rate was 0.53 nm s${}^{-1}$. The deposition rate was precisely measured in independent experiments on various substrates. The thickness of deposited gold was verified by profilometer (AMBIOS XP-I) [46,47,48,49,50].

The samples were investigated using a ThermoFisher Scientific Scios 2 dual beam microscope. The equipment is built with an acceleration tube, a unique in-lens Trinity detector system, and a retarding-field option. Taking advantage of this setup, low voltage scanning electron microscopy (LVSEM) technique was applied to make high-resolution images of the different aerogel samples, as detailed in the next subsection. An acceleration voltage of 1–2 kV and 2–5 mm working distance was typically used. The investigation of the gold coated samples was carried out by using the same conditions as for the uncoated (pristine) ones. The samples were fixed on vacuum-resistant carbon tape. Fresh fracture surfaces were investigated far from the point of splitting the aerogel monolith.

Nitrogen adsorption-desorption porosimetry measurements were performed with a Quantachrome Nova 2200e surface area and porosity analyzer Quantachrome Instruments, Boynton Beach, FL, USA). All samples were degassed in vacuum at 60 ${}^{\circ }$C for 24 h before the measurements. Raw data was evaluated with the NovaWin 11.0 software (Quantachrome Instruments, Boynton Beach, FL, USA). Total surface area was calculated according to the Brunauer-Emmett-Teller (BET) model. Pore size distribution plots were constructed using the Barret-Joyner-Halenda (BJH) method.

### 2.3. Low Voltage Scanning Electron Microscopy (LVSEM)

The nanostructures of the aerogel samples were investigated with a Field Emission Scanning Electron Microscope (FESEM) [51,52]. Scanning electron microscopy operating at electron energies below 5 keV is usually termed Low Voltage Scanning Electron Microscopy (LVSEM) [53]. The advantages of low acceleration voltage derive directly from the energy dependence of the electron-specimen interactions.

The penetration depth of the impinging electrons decreases with decreasing energy due to the reduced electron range, that is, the excitation volume shrinks in the specimen. The secondary electron (SE) yield increases because of the reduced electron range. The SEs that are generated near the surface can easily escape, which increases the SE yield. Because of the increasing SE yield, there should be a critical acceleration voltage for a given specimen where the amount of the incoming and the emitted electrons are balanced, and consequently, the specimen current equals to zero. This means that at this particular electron energy, no electric conductivity of the specimen is required. Ideally, imaging of electric insulators without conductive coating becomes possible. Further considerations on LVSEM technology are given in Supplementary Materials.

## 3. Results and Discussion

Selected key structural parameters of the set of inorganic, biopolymer, organic and hybrid aerogels investigated in this study are complied in Table 1. The results of the N${}_{2}$ gas porosimetry characterization of the different types of aerogels are summarized in the form of specific surface area (*S*_BET_), mean pore size and total pore volume. The complete porosimetry reports are given in the Supplementary Materials. It is clearly seen from Table 1, that the studied aerogels are significantly different from each other not only regarding their structural materials, but also in the sense of very distinct morphologies.

**Figure 1:**
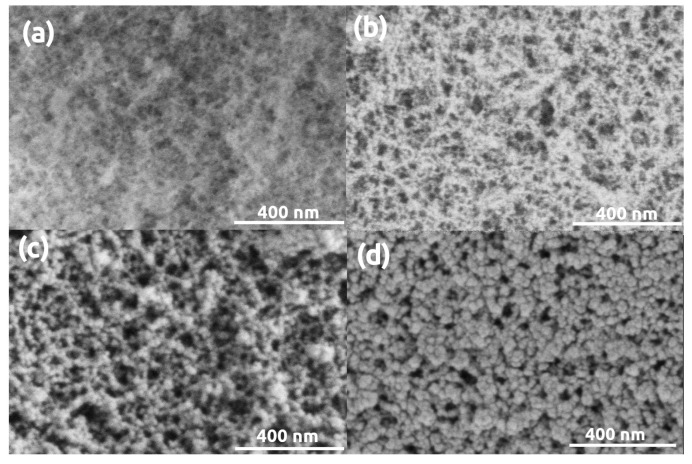
Low Voltage Scanning Electron Microscopy (LVSEM) images of silica aerogel samples. (**a**) Pristine, uncoated. (**b**–**d**) Sputter coated with 5 nm, 16 nm, 32 nm thick Au layers, respectively.

**Figure 2:**
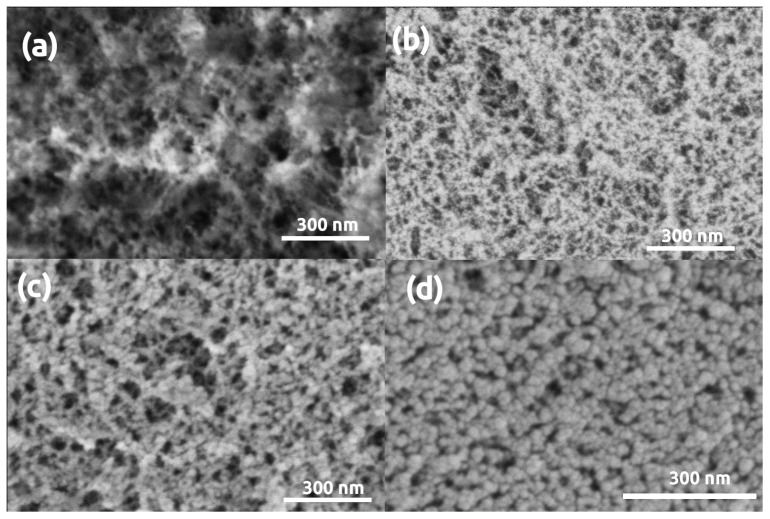
LVSEM images of silica-casein aerogel samples. (**a**) Pristine, uncoated. (**b**–**d**) Sputter coated with 5 nm, 16 nm, 32 nm thick Au layers, respectively.

**Figure 3:**
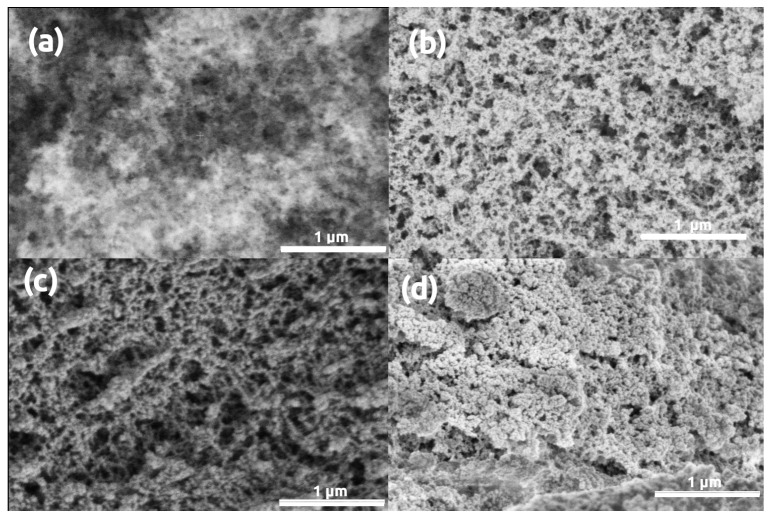
LVSEM images of silica-gelatin aerogel samples. (**a**) Pristine, uncoated. (**b**–**d**) Sputter coated with 5 nm, 16 nm, 32 nm thick Au layers, respectively.

Microscopy images of pristine (uncoated) and gold coated silica aerogel samples are presented in Figure 1. Sputtering even a thin, 5 nm gold layer results in the formation of artificial structural elements on the aerogel surface, and a 16 nm thick layer significantly modifies the morphology of silica aerogel. The initial snowflake-like network of pristine silica aerogel disappears under the Au coating [54]. Globular Au nanoparticles similar in size to the primary silica globules develop on the surface after sputtering 5 nm thick gold (Figure 1b). Following a second round of sputtering, the Au particles are considerably larger due to nucleation, and they completely hide the pristine structure of silica aerogel (Figure 1c) [46,47,48]. The same phenomenon was observed in the case of other silica-based aerogels, such as silica-casein and silica-gelatin hybrids (Figure 2 and Figure 3).

**Figure 4:**
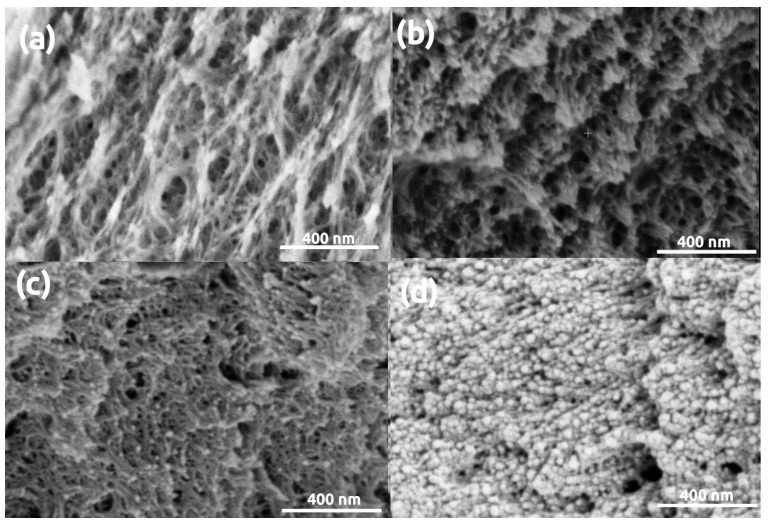
LVSEM images of Ca-alginate samples. (**a**) Pristine, uncoated. (**b**–**d**) Sputter coated with 5 nm, 16 nm, 32 nm thick Au layers, respectively.

Figure 4 shows representative LVSEM images of pristine and gold coated Ca-alginate aerogel samples. It is clearly seen that the original nanostructure of Ca-alginate aerogel is drastically altered by the sputtered gold layer. The original open framework of the Ca-alginate aerogel is composed of short fibrils of 20–25 nm of thickness. Coating by 5 nm thick gold layer results in the appearance of globular Au nanoparticles on the junction of the fibrils. After a second round of sputtering, the concentration and the size of the nanoparticles increase, and the structure of the pristine Ca-alginate aerogel is completely hidden.

**Figure 5:**
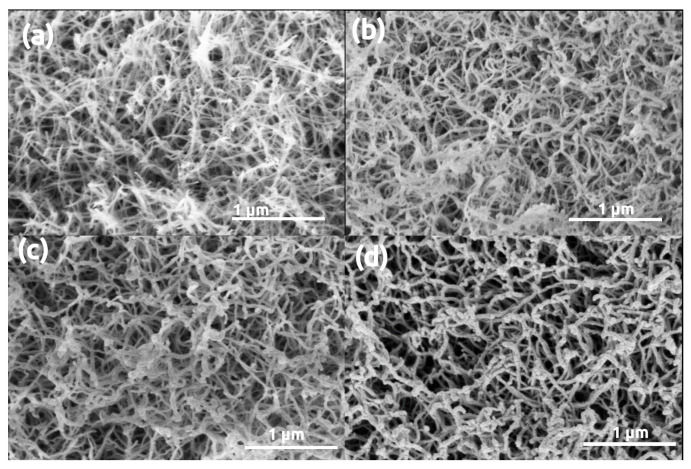
LVSEM images of polyimide aerogel samples. (**a**) Pristine, uncoated. (**b**–**d**) Sputter coated with 5 nm, 16 nm, 32 nm thick Au layers, respectively.

The morphologies of aramid polymer (polyimide and polyamide) aerogels are distinct from those of silica and biopolymers. Representative LVSEM images of polyimide aerogel samples are shown in Figure 5. The pristine polyimide aerogel is a loose network of polymer fibrils (struts) of ca. 28 nm thickness. After gold sputtering, the apparent thickness of fibrils significantly increases. Sputtering 16 nm coating causes the escalation of this phenomenon, that is, the apparent diameter of the fibrils doubles to ca. 50 nm.

**Figure 6:**
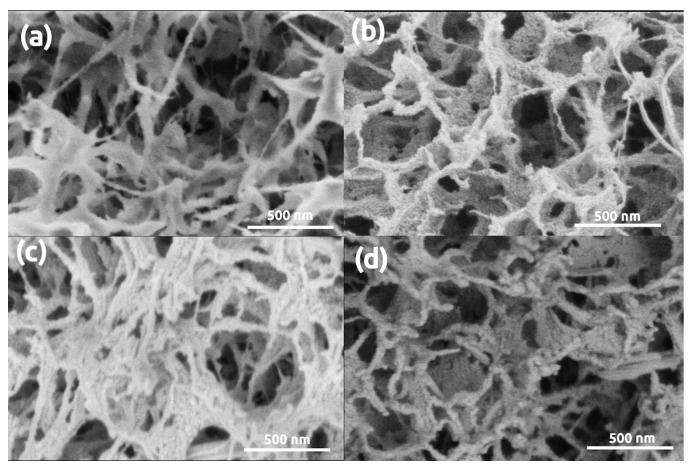
LVSEM images of polyamide aerogel samples. (**a**) Pristine, uncoated. (**b**–**d**) Sputter coated with 5 nm, 16 nm, 32 nm thick Au layers, respectively.

Representative LVSEM images of polyamide aerogel samples are shown in Figure 6. The three-dimensional structure of polyamide aerogel is composed of separated flat polymer strands that vary in size from a few nanometers to 100 nm. Gold deposition causes significant changes in the morphology, that is, small Au nanoparticles appear on the surfaces and on the edges of the strands. The deposition of thick gold coating causes the aggregation of the polymer strands and the formation of Au islands that eventually cover the whole polymer surface. It is notable that the original polymer strands are flat, while the covered strands are cylindrical.

**Figure 7:**
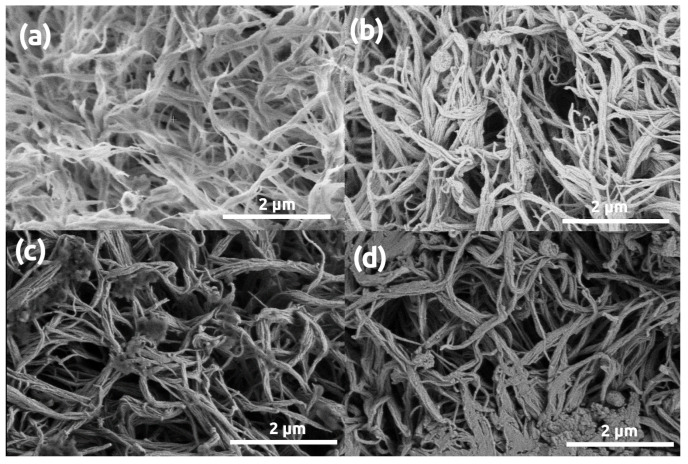
LVSEM images of polyamide-Ca(II) aerogel samples. (**a**) Pristine, uncoated. (**b**–**d**) Sputter coated with 5 nm, 16 nm, 32 nm thick Au layers, respectively.

The Ca(II) crosslinked polyamide aerogel shows unique morphological changes as a result of gold sputtering (Figure 7). In the case of the Ca(II) crosslinked polyamide aerogel, the formation of Au nanoparticles does not take place, instead the polymer strands are uniformly covered by a continuous layer of Au even when sputtering a thin 5 nm gold coating. Eventually, the polymer strands aggregate due to their coverage by gold similarly to the behavior of the polyamide aerogel.

The SEM images of the polyimide, polyamide and polyamide-Ca(II) aerogels (Figure 5, Figure 6 and Figure 7) were evaluated using image analysis. Approximately 20 manual measurements were performed on the images of the pristine and the coated samples in order to determine the thickness of the primary fibrils and pore size. The results are given in a tabulated form in Supplementary Materials. The numerical results are in very good agreement with the visual observations made on the SEM images. It is evident that fiber diameter dramatically increases as a consequence of gold coating in all polymer aerogels. Furthermore, pore sizes are dramatically altered in the case of the polyimide and polyamide-Ca(II) aerogels.

**Figure 8:**
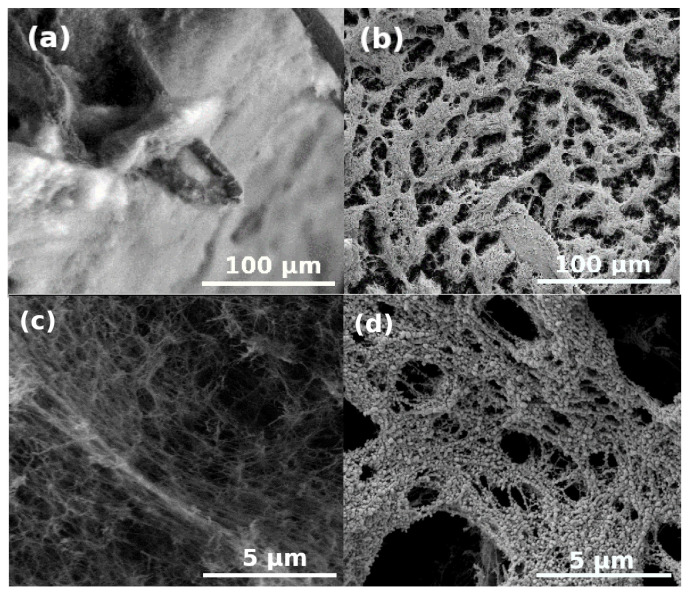
LVSEM images of cellulose aerogel samples. (**a**,**c**) Pristine, uncoated. (**b**,**d**) Plasma coated for 2.5 min.

Images of the cellulose aerogel sample 1.0%CL/1.0%CL-P are shown in Figure 8a before and after Figure 8b plasma coating. The image in Figure 8c was obtained in low vacuum (70 Pa) and it depicts the pristine sample. The image in Figure 8d was obtained in high vacuum and it depicts the coated sample. The comparison of the images reveals the partial destruction of the interconnected regenerated cellulose network and the formation of globular Au particles on the surface caused by the coating procedure. The morphology of these cellulose aerogels have been investigated by small-angle X-ray scattering (SAXS). The results were reported in our previous publication [55]. The diameter of the fibrillar (cylindrical) network forming primary particles is ca. 4 nm in the pristine aerogel. In contrast, the size of the globular units visible in the LVSEM images of the coated samples exceed 50 nm. Thus, the significant discrepancy between the morphologies derived from the SAXS results and visible on the LVSEM images of the coated sample clearly show the detrimental effect of gold sputtering.

In general, the observed morphological alterations of the very different types of aerogels are evidently caused by the sputtering of a metallic gold layer on their surfaces [46,47,48].

**Figure 9:**
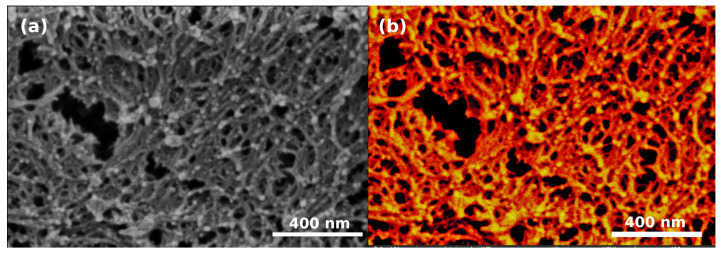
Images showing the combination of the as-obtained in-lens secondary electron (SE) and back scattered electron (BSE) signals of the 32 nm sputter coated Ca-alginate sample. The contrast correlates with atomic number: brighter regions correspond to higher atomic numbers. The aerogel consists of low atomic number elements, the bright spots are Au particles. The two images are identical, displayed in different color planes: (**a**) black/white and (**b**) red/white.

The characteristic size of the new morphological features formed on the aerogels after Au sputtering is a few tens of nanometers. The interaction volume for energy dispersive X-ray (EDX) analysis is much larger than the dimensions of these objects, because the penetration depth of electrons at 4 kV (necessary overvoltage to generate an EDX signal from Au) is ca. 160 nm according to Monte Carlo simulation performed with CASINO software [56]. Therefore, EDX mapping is not possible in the required resolution. In order to illustrate the distribution and localization of elements on a gold sputtered Ca-alginate aerogel sample surface, a SEM image is presented in Figure 9 that is a combination of the as-obtained in-lens SE and BSE signals. This image shows atomic number contrast: the brighter regions correspond to higher atomic numbers. Since the aerogel consists mainly of low atomic number elements (C, O, H, Ca) the bright globules in Figure 9 are Au particles.

Gold atoms condense to the surface during the deposition and form clusters of Au nanoparticles, which is driven by the reduction of the surface energy of the system. The deposition of thin metallic films could take place by different mechanisms (e.g., Volmer-Weber, Frank-Van der Merwe or Stranski-Krastanov models) and strongly depends on the substrate as well as the deposition conditions (pressure, atmosphere, temperature, gas flow, applied power, chemical composition, nanostructure and purity of the target material) [57]. Barna and Adamik reported a general model of film growth [58]. In brief, the process starts with the nucleation and island growth of the sputtered material which is followed by the coalescence of islands and the formation of polycrystalline islands. Due to the ripening of the formed islands, an even film forms and the thickness of the thin layer increases. It has been reported earlier that the deposition of gold on the surfaces of non-conducting aerogels results in the formation of porous gold films with similar structures to those of the substrate aerogels. It is reasonable to assume that the phenomenon observed in the present study is of the same origin as the one resulting in the formation of these porous gold films [59]. Finally, ion-bombardment during sputtering could also have a significant effect on the structures of aerogels [60].

## 4. Conclusions

Experimental evidence is presented in this work unambiguously proving that gold sputtering changes the native morphologies of several different types of non-conducting nanostructured aerogels. The results are of considerable significance since the application of gold sputtering is part of the routine of scanning electron microscopy (SEM) imaging; however, this practice can lead to the misinterpretation of the morphologies of the nanostructured samples.

The structures of silica-based aerogels are dramatically altered when a sputtered conductive Au layer is deposited on them to facilitate SEM imaging. The original snowflake-like three-dimensional network of silica-based aerogels is covered by globular Au nanoparticles. The original fibrillar building blocks of the Ca-alginate backbone can erroneously be misinterpreted as globules when a thick layer of gold is sputtered on the sample. In the case of aramid polymer aerogels, the initial morphologies of polymer strands are still visible after gold sputtering, but the diameter of the strands can increase significantly, even by a factor of 2. The fine-structure of cellulose aerogel is partially destroyed as a consequence of gold sputtering.

The utilization of low voltage scanning electron microscopy (LVSEM) is strongly advised for investigating non-conducting aerogels, because this technique enables the acquisition of high-resolution images of representative pristine (uncoated) samples. These erroneous conclusions regarding the nanostructures and morphologies of aerogels evidently falsify characterization efforts, and ultimately falsify structure-property relationship theories. Therefore, the utilization of low voltage scanning electron microscopy (LVSEM) is strongly advised for investigating non-conducting aerogels, because this technique enables the acquisition of high-resolution images of representative pristine (uncoated) samples.

## Figures and Tables

**Table 1:** Structural parameters derived from N${}_{2}$ gas adsorption-desorption porosimetry data.

Parameter	Silica	Silica- Gelatin	Silica- Casein	Ca- Alginate	Poly- Imide	Poly- Amide	Poly- Amide (Ca(II))	Method
Specific
surface area (m${}^{2}$ g${}^{-1}$)	898	742	750	544	297	245	251	BET
Mean pore
size (nm)	15	14	17	42	3.8	3.4	4.3	BJH
Total pore
volume (cm${}^{3}$ g${}^{-1}$)	7.5	3.2	3.2	7.8	0.8	0.8	0.7	BJH
C-constant	109	62	71	-	55	45	59	BET

## Data Availability

The data presented in this study are available in the main body of the article and in Appendices A and B. Additional data presented in this study are available on request from the corresponding author.

## Supplementary Materials

The following are available online at https://www.mdpi.com/2073-4360/13/4/588/s1, Additional considerations on microscopy theory and experiment; experimental details on aerogel preparation; nitrogen porosimetry reports. Results of image analysis of LVSEM pictures of pristine and sputter coated polyimide, polyamide, polyamide-Ca(II) aerogel samples presented in Figure 5, Figure 6 and Figure 7.
