# Supplementary material for: False Morphology of Aerogels Caused by Gold Coating for SEM Imaging
Source: Polymers (Basel). 2021 Feb 16;13(4):588. doi: 10.3390/polym13040588 (PMC7919642; doi:10.3390/polym13040588)
Supplement: Supplementary file 1 [file polymers-13-00588-s001.zip › _Appendices_A-B/polymers_1083760_appendix_A.pdf]

Article

Appendix A for "False Morphology of Aerogels Caused by Gold Coating for SEM Imaging"

Laura Juhász[1,2], Krisztián Moldován[3,4], Pavel Gurikov[5], Falk Liebner[6], István Fábián[3], József Kalmár[3] ‡ * and Csaba Cserháti[1] ‡ *

1 Department of Solid State Physics, University of Debrecen, Egyetem sqr. 1, Debrecen, H-4032 Hungary
2 Doctoral School of Physics, University of Debrecen, Egyetem sqr. 1, Debrecen, H-4032 Hungary
3 Department of Inorganic and Analytical Chemistry, MTA-DE Redox and Homogeneous Catalytic Reaction Mechanisms Research Group, University of Debrecen, Egyetem sqr. 1, Debrecen, H-4032 Hungary
4 Doctoral School of Chemistry, University of Debrecen, Egyetem sqr. 1, Debrecen, H-4032 Hungary
5 Laboratory for Development and Modelling of Novel Nanoporous Materials, Hamburg University of Technology, Eißendorfer Straße 38, 21073 Hamburg, Germany
6 University of Natural Resources and Life Sciences, Vienna (BOKU), Institute for Chemistry of Renewable Resources, Konrad-Lorenz-Straße 24, A-3430 Tulln, Austria
* Correspondence: cserhati.csaba@science.unideb.hu; Tel.: +36 52 316 073, kalmar.jozsef@science.unideb.hu, Tel.: +36 52 512 900

‡ - These authors contributed equally to the work.

Version January 4, 2021 submitted to Nanomaterials

1. Experimental

Aerogel synthesis

Silica aerogel [1]

Materials: Methanol (reagent grade), acetone (reagent grade), 25 wt.% ammonia solution (reagent grade) and tetramethyl orthosilicate (TMOS) were obtained from Sigma-Aldrich. All reagents were used without further purification.

Aerogel preparation: Two solutions (A and B) were prepared. Solution A was made from TMOS dissolved in methanol. Solution B contained methanol, distilled water and aqueous ammonia solution. The two solutions were mixed under stirring, then poured into a plastic mold. After 24 h, the alcogel was transferred into a perforated frame and aged in methanol. After 24 h, the sample was soaked in a mixture of acetone-methanol for another 24 h, and this step was repeated by increasing the acetone content every day. Finally, the sample was stored in acetone, and the gel was dried under supercritical conditions, as described previously. [1]

Silica-gelatin aerogel of 4 wt.% gelatin [2,3]

Materials: Household gelatin was obtained from Dr. Oetker. $(NH_4)_2CO_3$ were purchased from Fluka. All reagents were used without further purification.

Aerogel preparation: Gelatin and $(NH_4)_2CO_3$ were dissolved in hot water to give solution A. After cooling to room temperature, solution B containing TMOS and methanol was added to solution A under stirring. The mixture was poured in a cylindrical plastic mold for gelation. After 24 h, the alcogel was removed from the mold and placed into a perforated aluminum container for solvent exchange. First, the sample was soaked in methanol for 24 h to remove water. Next, methanol was replaced by acetone in four 24 h soaking steps, and acetone was replaced 2 more times after 24 h

soaking. Finally, acetone was extracted with liquid CO_2, then the gel was dried with supercritical CO_2, as described previously. [2,3]

Silica-casein aerogel of 28 wt.% casein [4]

Materials: The synthesis of the hybrid aerogels was realized from "MPI 85" casein powder, which was provided by the Hungarian Dairy Research Institute Ltd. (Mosonmagyaróvár, Hungary).

Aerogel preparation: In solution A, TMOS was dissolved in methanol. Solution B contained aqueous ammonia solution, water and methanol. In solution C, different quantities of the MPI 85 casein powder were completely dispersed in water. Solution B was added to solution A in a beaker during constant stirring. After a few minutes of mixing, solution C was also added. The sol was transferred into a plastic mold. The gel was kept in the mold for 3 days for completing the gelation. The gel was placed into a perforated aluminum container. For two days the gel was kept in methanol, for another 2 days it was in acetone: methanol (1:1) solution, and after that, it was kept in acetone for a week. The acetone was refreshed 3 times during this period. The gel was dried with supercritical CO_2, as described previously. [4]

Ca-alginate aerogel [5]

Materials: Sodium alginate was purchased from Sigma life science (catalogue no. 71238), $CaCl_2$ was purchased from Th. Geyer GmbH & Co. KG, Lohmar, Germany and used without any further purification.

Aerogel preparation: Spherical Ca-alginate aerogel beads of ca. 0.2 mm were synthesized by using the jet cutting method as described previously. [5,6] Briefly, 1 wt.% aqueous solution of Na-alginate was dropped into $CaCl_2$ solution (20 g/L) using a commercial JetCutter (Type S from geniaLab GmbH, Braunschweig, Germany). The gelation bath was moderately stirred in order to ensure the formation of spherical beads and to prevent their aggregation. After gelation, the gel beads were subjected to multiple step solvent exchange. The drying procedure was initiated only when the ethanol content reached min. 98.5 wt.% in order to ensure single phase conditions during subsequent supercritical drying. The gel beads were dried by extraction of ethanol with a continuous flow of supercritical CO_2 in a high-pressure autoclave, as described previously [5].

Polyimide aerogel [7]

Materials: Acetic anhydride (AA), triethylamine (TEA), 1,3,5-benzenetricarbonyl trichloride (BTC), N-methylpyrrolidinone (NMP), 2,2′-dimethylbenzidine (DMBZ) and biphenyl-3,3′,4,4′-tetracarboxylic dianhydride (BPDA) were purchased from Sigma-Aldrich. All reagents were used without further purification.

Aerogel preparation: BPDA was added to a solution of NMP and DMBZ, and the resulting mixture was stirred until the solid materials dissolved. Then AA and TEA was added to the mixture. Another solution was prepared by dissolving BTC in NMP. This solution was added to the first solution under stirring. After mixing, the solution was poured into the mold and the gel was aged for 24 hours. The gel was soaked in a solution of 75 % NMP in acetone. Afterwards, the solvent was first replaced by a solution of 25 % NMP in acetone, and finally three times by 100 % acetone. The gel was dried with supercritical CO_2 in a pumpless system, described in a previous publication [8].

Polyamide aerogel [9]

Materials: Isophthaloyl chloride (IPC), *m*-phenylene diamine (*m*-PDA) and 1,3,5-benzenetricarbonyl trichloride (BTC) were purchased from Sigma-Aldrich. All reagents were used without further purification.

Aerogel preparation: A solution of *m*-PDA in NMP was cooled to 5 °C using an ice water bath. IPC was added in one portion to the cooled solution. BTC was added and the mixture was vigorously stirred for 5 min before being poured into molds. After aging overnight at room temperature, the

monoliths were removed from the molds and placed in ethanol. This was followed by seven solvent exchange steps in 24 h intervals. The gel was dried with supercritical CO_2 in a pumpless system, described in a previous publication. [8]

Ca(II) crosslinked polyamide aerogel [10]

Materials: Calcium chloride ($CaCl_2$), terephthaloyl chloride (TPC) and *p*-phenylenediamine (*p*-PDA) were purchased from Sigma-Aldrich. All reagents were used without further purification.

Aerogel preparation: A solution of $CaCl_2$ and *p*-PDA in NMP was prepared and cooled to 0 °C using an ice-water bath. Then solid TPC was added to the mixture and a transparent yellow solution formed. After stirring, the suspension was poured into an aluminum mold and kept overnight at room temperature. The resulting yellow cylindrical monolith was removed from the mold and soak in ethanol for 5 days. The solvent was replaced with fresh ethanol each day. The gel was dried with supercritical CO_2 in a pumpless system, described in a previous publication. [8]

Cellulose aerogel [11]

Cellulose aerogels were prepared from cotton linters (CL) and phosphorylated cotton linters (CL-P). Characterization of the starting material, phosphorylation of cellulose, the preparation of the cellulose solvent system were reported previously. [11] Aerogel preparation was accomplished by cellulose dissolution in the TBAF·H_2O/DMSO solvent system, and coagulation by addition of ethanol followed by solvent exchange and drying in supercritical CO_2. The detailed recipe and the process parameters are given in [11].

2. Low Voltage Scanning Electron Microscopy (LVSEM)

To achieve the correct value of the critical acceleration voltage, in the optical system of a modern FESEM instrument, a combination of magnetic and electrostatic lenses (LVSEM) are used as the alternatives to the traditionally used magnetic lenses. A microscope equipped with a combined objective lens permits non-constant beam energy along the column. The beam electrons pass the column with high energy and are decelerated to low energy in the immersion electrostatic lens. The consequences of that are first, the magnitude of the aberrations of immersion electrostatic lenses corresponds to the high energy at the entrance side, second, the high electron energy in the column is advantageous because the gun brightness increases with electron energy, and electromagnetic stray fields result in less deterioration of the electron beam at high energy. The aberration coefficients of the combined magnetic–electrostatic objective lens can be very low compared to the traditional magnetic lenses. Note, that very low landing energies of the electrons can be realized with a retarding-field SEM. Applying these techniques guarantee small probe size and high signal-to-noise ratio [12]. These features enable the imaging of non-conducting samples in their pristine forms in high resolution.

Those beam electrons that are elastically scattered and emerge from the specimen as BSEs, also generate secondary electrons within the escape depth. The origin of these secondary electrons is far from the point of incidence of the beam, thus these deliver low-resolution information. These secondary electrons can be excluded from the detection process if a short working distance (< 2 mm) is chosen.

In order to detect signals with such a small working distance a special detector strategies are required. Most of the modern FESEM instruments feature some form of in-lens or through-the-lens detection system that has the ability to separate and collect SEs, back scattered electrons (BSEs), or a mixture of both types of signals.

1. Kalmár, J.; Kéri, M.; Erdei, Z.; Bányai, I.; Lázár, I.; Lente, G.; Fábián, I. The Pore Network and the Adsorption Characteristics of Mesoporous Silica Aerogel: Adsorption, Kinetics on a Timescale of Seconds. *RSC Adv.* **2015**, *5*, 107237–107246. doi:10.1039/C5RA21353C.
2. Veres, P.; Kéri, M.; Bányai, I.; Lázár, I.; Fábián, I.; Domingo, C.; Kalmár, J. Mechanism of Drug Release from Silica-gelatin Aerogel-Relationship between Matrix Structure and Release Kinetics. *Colloids Surf., B* **2017**, *152*, 229 – 237. doi:https://doi.org/10.1016/j.colsurfb.2017.01.019.
3. Kéri, M.; Forgács, A.; Papp, V.; Banyai, I.; Veres, P.; Len, A.; Dudás, Z.; Fábián, I.; Kalmár, J. Gelatin Content Governs Hydration Induced Structural Changes in Silica-gelatin Hybrid Aerogels – Implications in Drug Delivery. *Acta Biomater.* **2020**, *105*, 131–145. doi:10.1016/j.actbio.2020.01.016.
4. Lázár, I.; Forgács, A.; Horváth, A.; Király, G.; Nagy, G.; Len, A.; Dudás, Z.; Papp, V.; Balogh, Z.; Moldován, K.; Juhász, L.; Cserháti, C.; Szántó, Z.; Fábián, I.; Kalmár, J. Mechanism of Hydration of Biocompatible Silica-casein Aerogels Probed by NMR and SANS Reveal Backbone Rigidity. *Appl. Surf. Sci.* **2020**, *531*, 147232. doi:https://doi.org/10.1016/j.apsusc.2020.147232.
5. Preibisch, I.; Niemeyer, P.; Yusufoglu, Y.; Gurikov, P.; Milow, B.; Smirnova, I. Polysaccharide-Based Aerogel Bead Production via Jet Cutting Method. *Materials* **2018**, *11*, 1287. doi:10.3390/ma11081287.
6. Ganesan, K.; Budtova, T.; Ratke, L.; Gurikov, P.; Baudron, V.; Preibisch, I.; Niemeyer, P.; Smirnova, I.; Milow, B. Review on the Production of Polysaccharide Aerogel Particles. *Materials* **2018**, *11*, 2144. doi:10.3390/ma11112144.
7. Meador, M.A.B.; Alemán, C.R.; Hanson, K.; Ramirez, N.; Vivod, S.L.; Wilmoth, N.; McCorkle, L. Polyimide Aerogels with Amide Cross-Links: A Low Cost Alternative for Mechanically Strong Polymer Aerogels. *ACS Appl. Mater. Interfaces* **2015**, *7*, 1240–1249. doi:10.1021/am507268c.
8. Lázár, I.; Fábián, I. A Continuous Extraction and Pumpless Supercritical CO_2 Drying System for Laboratory-Scale Aerogel Production. *Gels* **2016**, *2*, 26. doi:10.3390/gels2040026.
9. Williams, J.C.; Meador, M.A.B.; McCorkle, L.; Mueller, C.; Wilmoth, N. Synthesis and Properties of Step-Growth Polyamide Aerogels Cross-linked with Triacid Chlorides. *Chem. Mater.* **2014**, *26*, 4163–4171. doi:10.1021/cm5012313.
10. Williams, J.C.; Nguyen, B.N.; McCorkle, L.; Scheiman, D.; Griffin, J.S.; Steiner, S.A.; Meador, M.A.B. Highly Porous, Rigid-Rod Polyamide Aerogels with Superior Mechanical Properties and Unusually High Thermal Conductivity. *ACS Appl. Mater. Interfaces* **2017**, *9*, 1801–1809. doi:10.1021/acsami.6b13100.
11. Schimper, C.B.; Pachschwoell, P.S.; Hettegger, H.; Neouze, M.A.; Nedelec, J.M.; Wendland, M.; Rosenau, T.; Liebner, F. Aerogels from Cellulose Phosphates of Low Degree of Substitution: A TBAF·H2O/DMSO Based Approach. *Molecules* **2020**, *25*, 1695. doi:10.3390/molecules25071695.
12. Erdman, N.; Bell, D.C.; Reichelt, R., Scanning Electron Microscopy. In *Springer Handbook of Microscopy*; Hawkes, P.; Spence, J.C., Eds.; Springer International Publishing: Cham, 2019; pp. 229–318. doi:10.1007/978-3-030-00069-1_5.

Publisher's Note: MDPI stays neutral with regard to jurisdictional claims in published maps and institutional affiliations.

© 2021 by the authors. Submitted to *Nanomaterials* for possible open access publication under the terms and conditions of the Creative Commons Attribution (CC BY) license (http://creativecommons.org/licenses/by/4.0/).

Silica aerogel

Isotherm * Linear

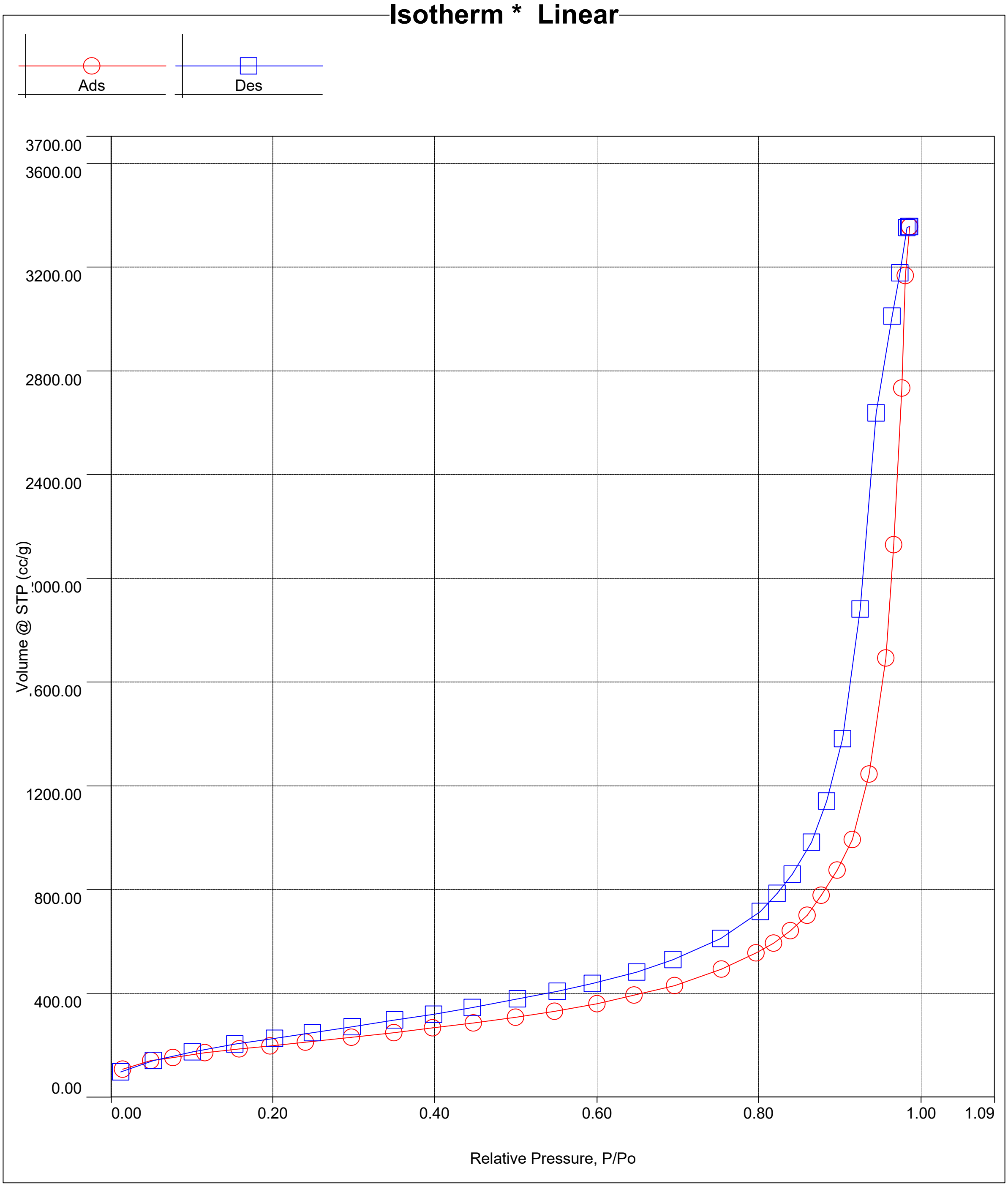

Quantachrome NovaWin - Data Acquisition and Reduction
for NOVA instruments
©1994-2010, Quantachrome Instruments
version 11.0

Quantachrome INSTRUMENTS
Optimizing particle performance

**Analysis**		**Report**	
**Operator:**AerogelLab	**Date:**2017/08/25	**Operator:**AerogelLab	**Date:**9/28/2020
**Sample ID:** P[illegible]	**Filename:**	C:\Users\User\Documents\Munka\SEM cikk\Porozimetria\P	

Isotherm * Log Scale

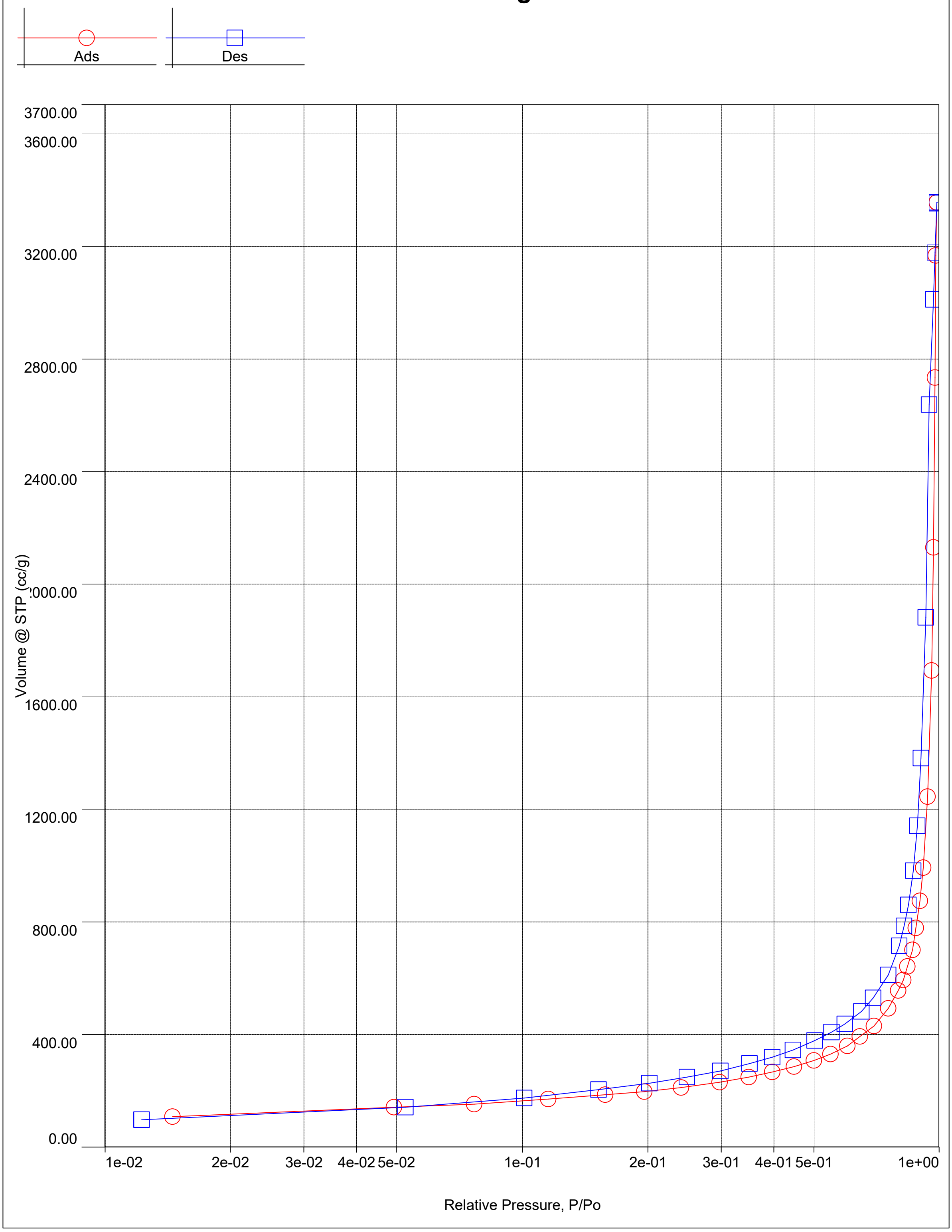

**Analysis**		**Report**	
**Operator:**AerogelLab	**Date:**2017/08/25	**Operator:**AerogelLab	**Date:**9/28/2020
**Sample ID:** P	**Filename:**	C:\Users\User\Documents\Munka\SEM cikk\Porozimetria\P	

Isotherm

Relative Pressure	Volume @ STP [cc/g]	Relative Pressure	Volume @ STP [cc/g]	Relative Pressure	Volume @ STP [cc/g]
1.45540e-02	108.2194	8.59391e-01	701.6637	8.22941e-01	784.4884
4.93930e-02	140.6369	8.77377e-01	780.0085	8.01492e-01	714.6162
7.65640e-02	155.1502	8.96079e-01	873.6975	7.52793e-01	613.3334
1.15609e-01	171.3006	9.15099e-01	995.0113	6.93847e-01	529.5925
1.58346e-01	187.0176	9.35615e-01	1243.5071	6.49108e-01	481.2757
1.96393e-01	199.4565	9.56442e-01	1694.5931	5.94238e-01	438.1250
2.40648e-01	214.0437	9.66118e-01	2130.4215	5.50691e-01	408.0417
2.97224e-01	232.3975	9.75859e-01	2730.9642	5.01537e-01	379.0505
3.48842e-01	249.4064	9.79950e-01	3166.7746	4.45857e-01	346.0070
3.97361e-01	267.9889	9.85565e-01	3355.4858	3.98076e-01	321.6872
4.47505e-01	287.2261	9.84322e-01	3355.2582	3.49991e-01	298.5350
4.99651e-01	308.1539	9.82696e-01	3350.3434	2.98149e-01	273.0877
5.47598e-01	332.7020	9.74060e-01	3178.2415	2.48640e-01	250.1214
5.99976e-01	359.9337	9.63979e-01	3008.2772	2.01965e-01	227.7140
6.45293e-01	393.0011	9.44511e-01	2636.0795	1.52644e-01	203.7132
6.95770e-01	430.2155	9.25007e-01	1883.1046	1.01004e-01	175.8068
7.53531e-01	493.9127	9.03429e-01	1380.1966	5.25150e-02	144.1124
7.95950e-01	555.4911	8.83903e-01	1141.4828	1.22850e-02	97.7589
8.18561e-01	593.3118	8.64536e-01	982.2632		
8.39265e-01	641.5597	8.40913e-01	859.1317		

Quantachrome INSTRUMENTS Optimizing particle performance

Analysis
Operator: AerogelLab **Date:** 2015/03/20
Sample ID: P **Filename:** C:\Users\User\Documents\Munka\SEM cikk\Porozimetria\P
Sample Desc: **Comment:**
Sample weight: 0.0283 g **Sample Volume:** 0 cc
Outgas Time: 11.0 hrs **OutgasTemp:** 50.0 C
Analysis gas: Nitrogen **Bath Temp:** 77.3 K
Press. Tolerance: 0.100/0.100 (ads/des) **Equil time:** 180/180 sec (ads/des) **Equil timeout:** 360/360 sec (ads/des)
Analysis Time: 1385.6 min **End of run:** 2015/03/20 18:24:13 **Instrument:** Nova Station A
Cell ID: 2 **F/W version:** 0.00

Report
Operator: AerogelLab **Date:** 9/24/2020

Data Reduction Parameters

Adsorbate Nitrogen **Temperature** 77.350K
Molec. Wt.: 28.013 g **Cross Section:** 16.200 Ų **Liquid Density:** 0.808 g/cc

MBET summary

Slope = 3.787
Intercept = 3.490e-02
Correlation coefficient, r = 0.999893
C constant= 108.575

Surface Area = 897.941 m²/g

Multi-Point BET Plot

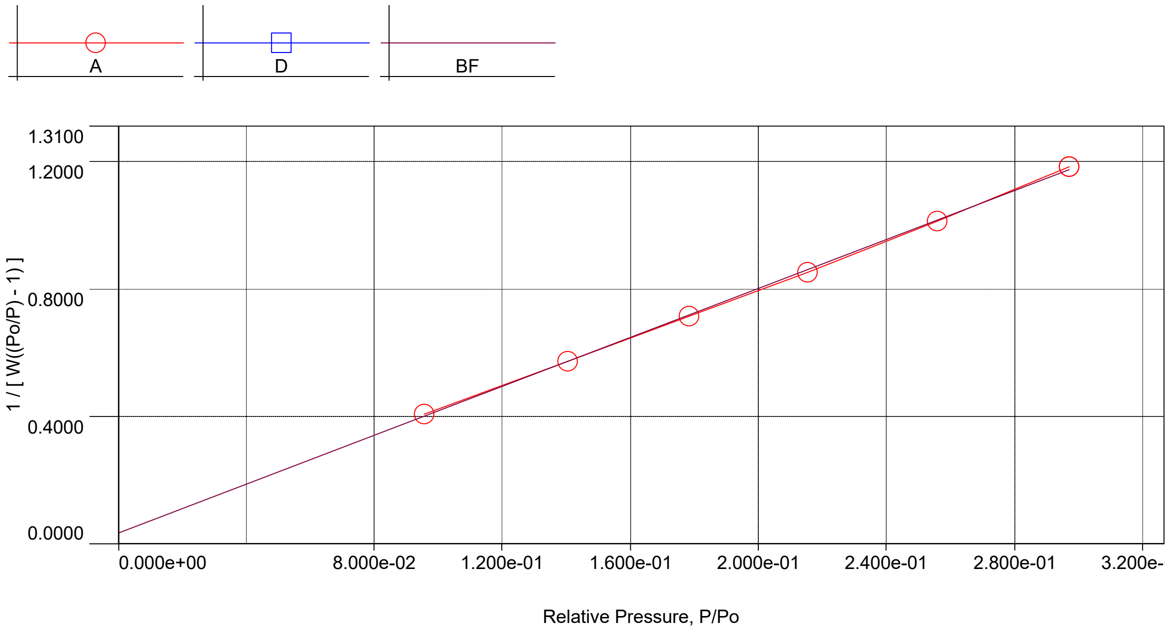

Multi-Point BET

Relative Pressure [P/Po]	Volume @ STP [cc/g]	1 / [W((Po/P) - 1)]	Relative Pressure [P/Po]	Volume @ STP [cc/g]	1 / [W((Po/P) - 1)]
9.55050e-02	207.5431	4.0706e-01	2.15245e-01	257.4815	8.5232e-01
1.40361e-01	227.9969	5.7300e-01	2.56026e-01	271.6151	1.0137e+00
1.78347e-01	243.2323	7.1401e-01	2.97108e-01	285.8777	1.1830e+00

Quantachrome NovaWin - Data Acquisition and Reduction
for NOVA instruments
©1994-2010, Quantachrome Instruments
version 11.0

Quantachrome INSTRUMENTS
Optimizing particle performance

**Analysis**				**Report**		
**Operator:** AerogelLab		**Date:** 2015/03/20		**Operator:** AerogelLab	**Date:** 9/24/2020	
**Sample ID:**	P	**Filename:**	C:\Users\User\Documents\Munka\SEM cikk\Porozimetria\P			
**Sample Desc:**		**Comment:**				
**Sample weight:**	0.0283 g	**Sample Volume:**	0 cc			
**Outgas Time:**	11.0 hrs	**OutgasTemp:**	50.0 C			
**Analysis gas:**	Nitrogen	**Bath Temp:**	77.3 K			
**Press. Tolerance:**	0.100/0.100 (ads/des)	**Equil time:**	180/180 sec (ads/des)	**Equil timeout:**	360/360 sec (ads/des)	
**Analysis Time:**	1385.6 min	**End of run:**	2015/03/20 18:24:13	**Instrument:**	Nova Station A	
**Cell ID:**	2			**F/W version:**	0.00	

Data Reduction Parameters

**t-Method**	**Calc. method:** de Boer				
**BJH/DH method**	**Moving pt. avg.:** off	**Ignoring P-tags below 0.35 P/Po**			
**Adsorbate**	**Nitrogen**	**Temperature**	77.350K		
	**Molec. Wt.:** 28.013 g	**Cross Section:**	16.200 Å	**Liquid Density:**	0.808 g/cc
	**Contact Angle:** 0.0 degrees	**Surf. Tension:**	8.850 erg/cm		

BJH method Desorption dV(log)

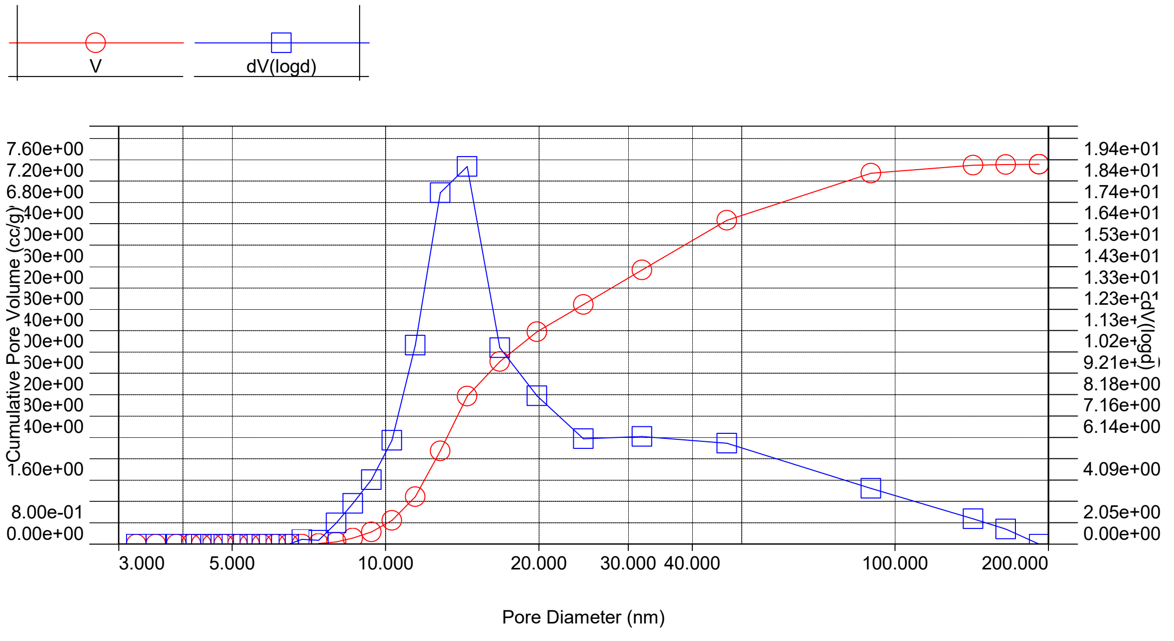

BJH desorption summary

**Surface Area =**	1398.323 m² /g
**Pore Volume =**	7.471 cc/g
**Pore Diameter Dv(d) =**	14.651 nm

Analysis
Operator: AerogelLab **Date:**2015/03/20
Sample ID: P **Filename:**

Report
Operator: AerogelLab **Date:**9/24/2020
C:\Users\User\Documents\Munka\SEM cikk\Porozimetria\P

BJH Pore Size Distribution Desorption

Diameter [nm]	Pore Volume [cc/g]	Pore Surf Area [m^2/g]	dV(d) [cc/nm/g]	dS(d) [m^2/nm/g]	dV(logd) [cc/g]	dS(logd) [cc/g]
3.2453	0.0000e+00	0.0000e+00	0.0000e+00	0.0000e+00	0.0000e+00	0.0000e+00
3.5462	0.0000e+00	0.0000e+00	0.0000e+00	0.0000e+00	0.0000e+00	0.0000e+00
3.8959	0.0000e+00	0.0000e+00	0.0000e+00	0.0000e+00	0.0000e+00	0.0000e+00
4.1624	0.0000e+00	0.0000e+00	0.0000e+00	0.0000e+00	0.0000e+00	0.0000e+00
4.3598	0.0000e+00	0.0000e+00	0.0000e+00	0.0000e+00	0.0000e+00	0.0000e+00
4.5905	0.0000e+00	0.0000e+00	0.0000e+00	0.0000e+00	0.0000e+00	0.0000e+00
4.8284	0.0000e+00	0.0000e+00	0.0000e+00	0.0000e+00	0.0000e+00	0.0000e+00
5.1232	0.0000e+00	0.0000e+00	0.0000e+00	0.0000e+00	0.0000e+00	0.0000e+00
5.4139	0.0000e+00	0.0000e+00	0.0000e+00	0.0000e+00	0.0000e+00	0.0000e+00
5.7228	0.0000e+00	0.0000e+00	0.0000e+00	0.0000e+00	0.0000e+00	0.0000e+00
6.0859	0.0000e+00	0.0000e+00	0.0000e+00	0.0000e+00	0.0000e+00	0.0000e+00
6.4693	0.0000e+00	0.0000e+00	0.0000e+00	0.0000e+00	0.0000e+00	0.0000e+00
6.8662	5.6543e-03	3.2940e+00	1.4449e-02	8.4174e+00	2.2837e-01	1.3304e+02
7.3928	1.2779e-02	7.1491e+00	1.0763e-02	5.8236e+00	1.8310e-01	9.9066e+01
8.0043	4.3530e-02	2.2516e+01	5.4829e-02	2.7400e+01	1.0101e+00	5.0478e+02
8.6555	1.1627e-01	5.6130e+01	9.8069e-02	4.5321e+01	1.9533e+00	9.0270e+02
9.4152	2.2748e-01	1.0338e+02	1.4300e-01	6.0754e+01	3.0984e+00	1.3163e+03
10.3128	4.4083e-01	1.8613e+02	2.0970e-01	8.1337e+01	4.9756e+00	1.9299e+03
11.4330	8.8410e-01	3.4122e+02	3.6242e-01	1.2680e+02	9.5318e+00	3.3348e+03
12.8021	1.7505e+00	6.1190e+02	5.7180e-01	1.7866e+02	1.6836e+01	5.2603e+03
14.5044	2.7754e+00	8.9455e+02	5.4247e-01	1.4960e+02	1.8091e+01	4.9892e+03
16.7691	3.4198e+00	1.0483e+03	2.4409e-01	5.8223e+01	9.4052e+00	2.2435e+03
19.8748	3.9764e+00	1.1603e+03	1.5585e-01	3.1367e+01	7.1132e+00	1.4316e+03
24.4846	4.4846e+00	1.2433e+03	8.9973e-02	1.4699e+01	5.0499e+00	8.2499e+02
31.8830	5.1317e+00	1.3245e+03	7.0737e-02	8.8745e+00	5.1572e+00	6.4701e+02
46.6529	6.0626e+00	1.4043e+03	4.5650e-02	3.9140e+00	4.8247e+00	4.1367e+02
89.5611	6.9485e+00	1.4439e+03	1.3541e-02	6.0475e-01	2.6634e+00	1.1895e+02
142.3293	7.0964e+00	1.4480e+03	3.6887e-03	1.0367e-01	1.2008e+00	3.3748e+01
165.1212	7.1069e+00	1.4483e+03	1.9077e-03	4.6214e-02	7.2526e-01	1.7569e+01
191.3929	7.1084e+00	1.4483e+03	3.3015e-05	6.9000e-04	1.4476e-02	3.0254e-01

Silica-gelatin aerogel

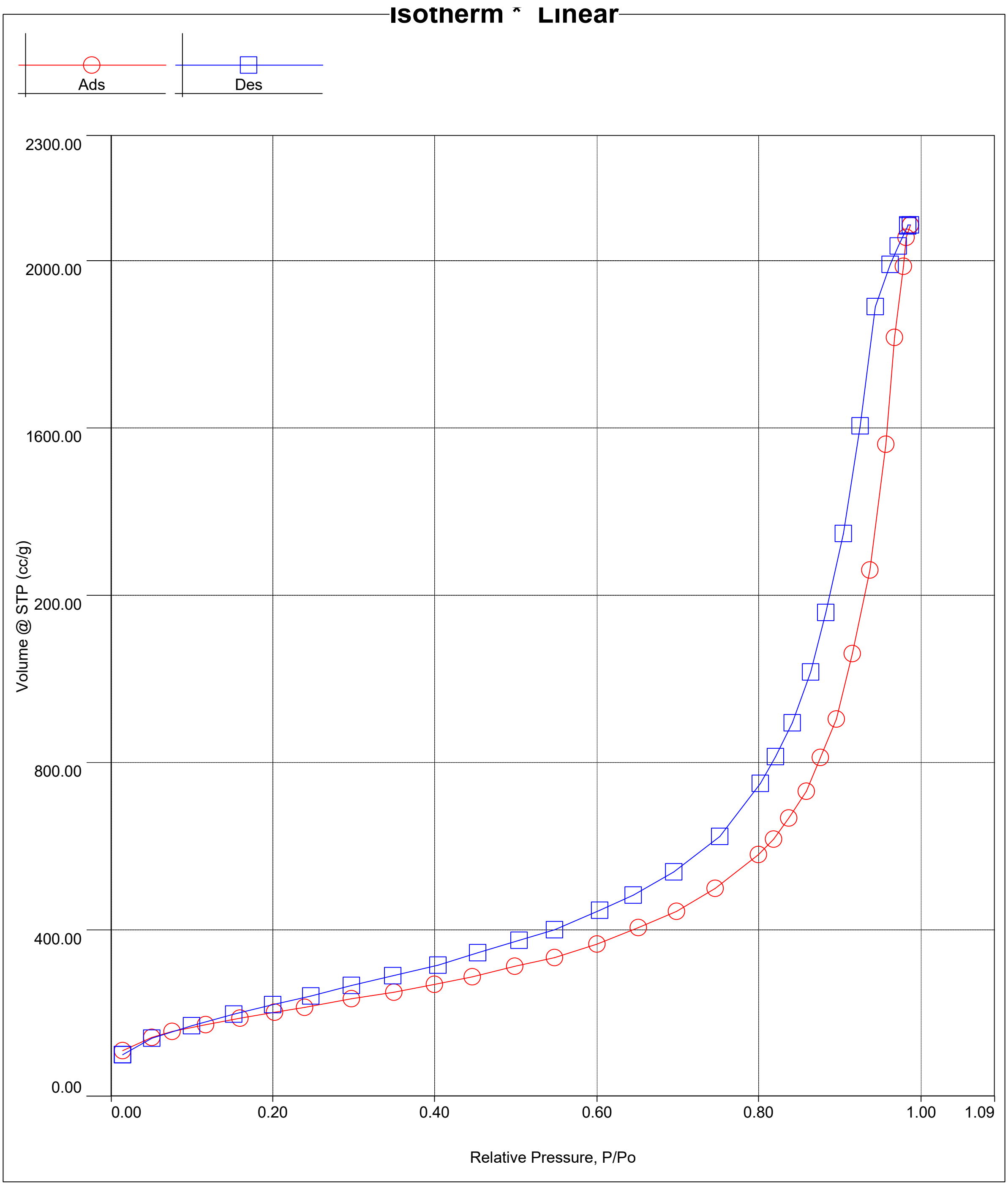

Quantachrome INSTRUMENTS Optimizing particle performance

Analysis
Operator: AerogelLab **Date:**2017/11/16
Sample ID: P 77 I **Filename:**

Report
Operator: AerogelLab **Date:**9/24/2020
C:\Users\User\Documents\Munka\SEM cikk\Porozimetria\P 77 I 125 1.qps

Isotherm * Log Scale

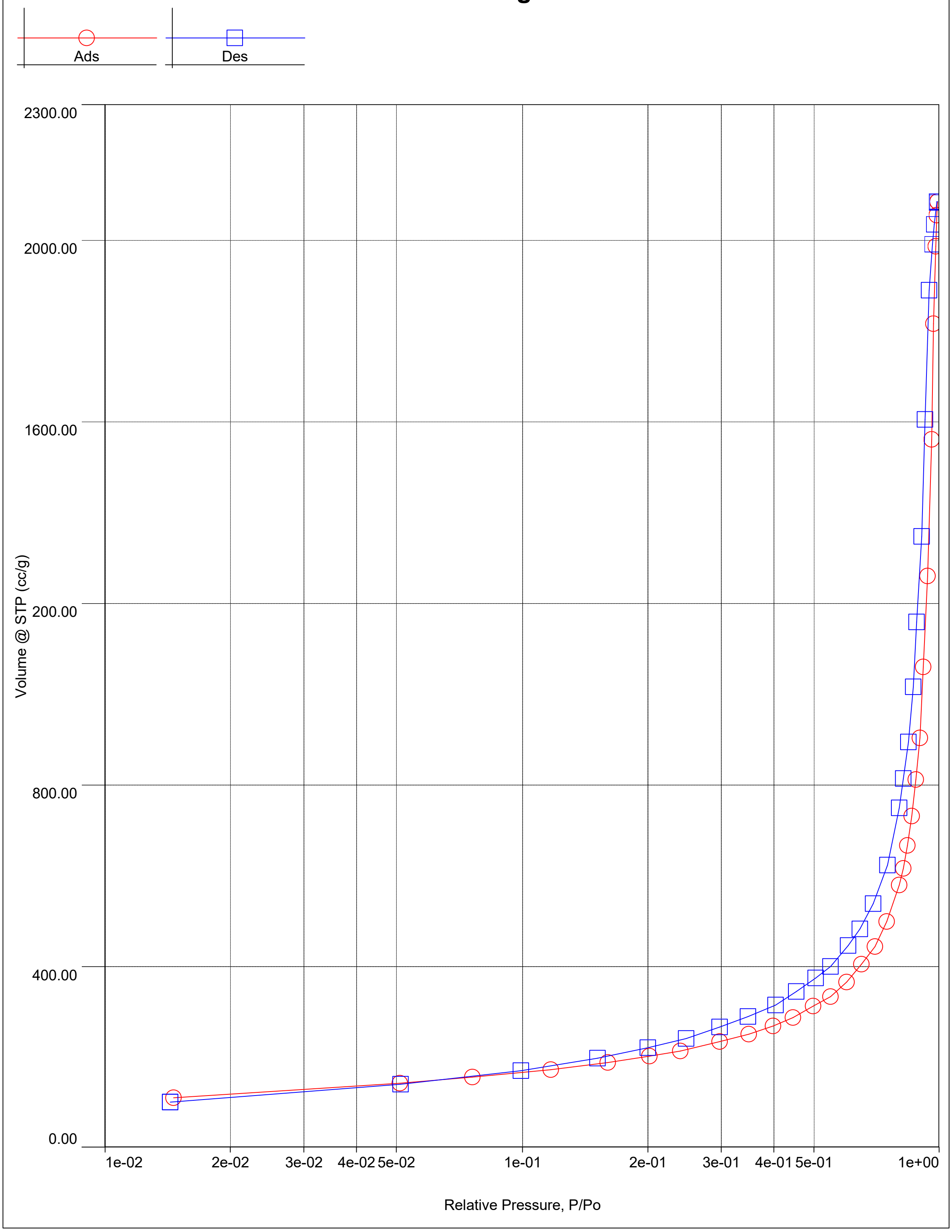

Analysis
Operator: AerogelLab **Date:**2017/11/16
Sample ID: P 77 I **Filename:**

Report
Operator: AerogelLab **Date:**9/24/2020
C:\Users\User\Documents\Munka\SEM cikk\Porozimetria\P 77 I 125 1.qps

Isotherm

Relative Pressure	Volume @ STP [cc/g]	Relative Pressure	Volume @ STP [cc/g]	Relative Pressure	Volume @ STP [cc/g]
1.46320e-02	109.8030	8.58522e-01	729.9420	8.20013e-01	812.7103
5.10010e-02	141.8523	8.75539e-01	810.3543	8.01639e-01	747.8360
7.60990e-02	154.6160	8.95573e-01	901.8319	7.51543e-01	622.2457
1.17029e-01	171.6371	9.15169e-01	1058.0374	6.94357e-01	537.5081
1.60071e-01	187.0141	9.36374e-01	1258.5065	6.45081e-01	481.5551
2.01870e-01	200.8426	9.56013e-01	1559.9928	6.03073e-01	445.5563
2.39718e-01	213.4057	9.66960e-01	1816.1033	5.47288e-01	399.6352
2.97428e-01	232.3094	9.78331e-01	1986.8203	5.04046e-01	372.8947
3.49010e-01	250.0419	9.81880e-01	2053.9636	4.53210e-01	343.0923
3.99164e-01	267.7248	9.86965e-01	2085.0164	4.04271e-01	314.4983
4.46408e-01	286.4422	9.84128e-01	2085.4690	3.47789e-01	287.5853
4.98414e-01	310.8411	9.83446e-01	2083.6421	2.97493e-01	264.7564
5.47856e-01	333.3697	9.71543e-01	2033.3622	2.46887e-01	240.5019
5.99791e-01	363.8025	9.62428e-01	1991.4053	2.00253e-01	219.6346
6.50957e-01	403.7951	9.43464e-01	1889.9878	1.51681e-01	195.6967
6.98301e-01	443.7594	9.24335e-01	1603.9860	9.93650e-02	169.0243
7.46076e-01	497.2603	9.04028e-01	1346.7617	5.10730e-02	138.1047
7.99699e-01	577.4813	8.82047e-01	1159.0985	1.43590e-02	99.4622
8.17894e-01	614.6000	8.63723e-01	1016.3821		
8.36132e-01	666.3063	8.40715e-01	894.4916		

Analysis
Operator: AerogelLab **Date:**2017/11/16
Sample ID: P 77 I **Filename:** C:\Users\User\Documents\Munka\SEM cikk\Porozimetria\P 77 I 125 1.qps
Sample Desc: szilika zselatin **Comment:**
Sample weight: 0.02054 g **Sample Volume:** 0 cc
Outgas Time: 24.0 hrs **OutgasTemp:** 100.0 C
Analysis gas: Nitrogen **Bath Temp:** 77.3 K
Press. Tolerance: 0.070/0.070 (ads/des) **Equil time:** 300/300 sec (ads/des) **Equil timeout:** 600/600 sec (ads/des)
Analysis Time: 1049.2 min **End of run:** 2017/11/16 2:39:01 **Instrument:** Nova Station B
Cell ID: 1 **F/W version:** 0.00

Report
Operator: AerogelLab **Date:**9/24/2020

Data Reduction Parameters

Adsorbate **Nitrogen** **Temperature** 77.350K
Molec. Wt.: 28.013 g **Cross Section:** 16.200 Ų **Liquid Density:** 0.808 g/cc

MBET summary

Slope = 4.615
Intercept = 7.632e-02
Correlation coefficient, r = 0.999994
C constant= 61.466

Surface Area = 742.398 m²/g

Multi-Point BET Plot

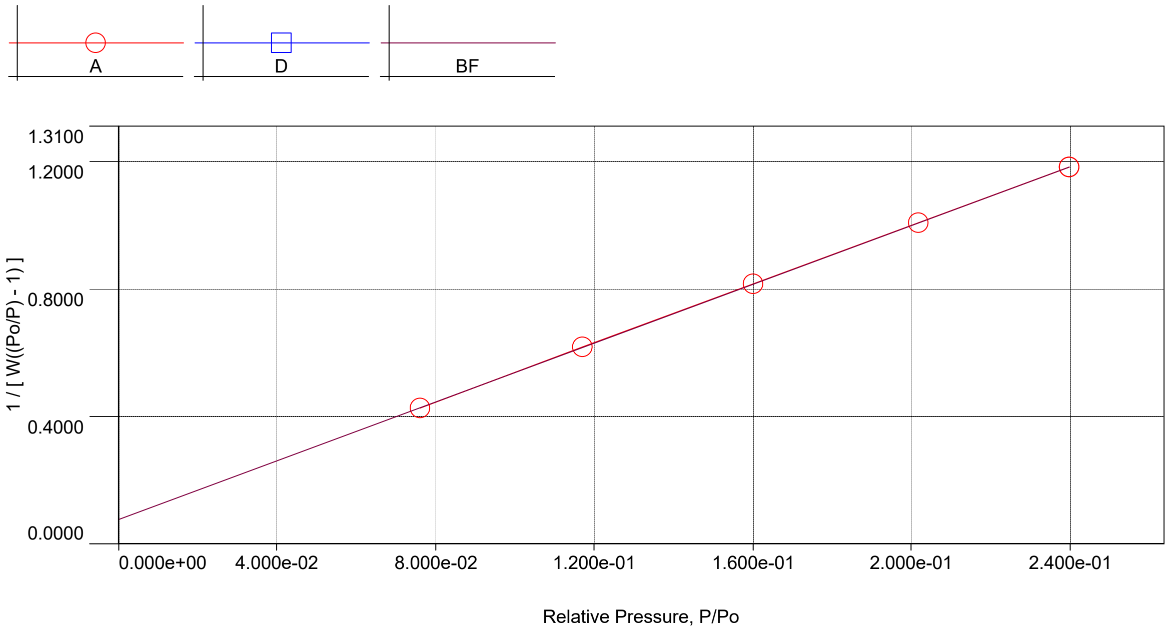

Multi-Point BET

Relative Pressure [P/Po]	Volume @ STP [cc/g]	1 / [W((Po/P) - 1)]
7.60990e-02	154.6160	4.2624e-01
1.17029e-01	171.6371	6.1785e-01
1.60071e-01	187.0141	8.1535e-01
2.01870e-01	200.8426	1.0076e+00
2.39718e-01	213.4057	1.1821e+00

Quantachrome INSTRUMENTS
Optimizing particle performance

**Analysis**					
**Operator:** AerogelLab	**Date:** 2017/11/16		**Report Operator:** AerogelLab	**Date:** 9/24/2020	
**Sample ID:**	P 77 I	**Filename:**	C:\Users\User\Documents\Munka\SEM cikk\Porozimetria\P 77 I 125 1.qps		
**Sample Desc:**	szilika zselatin	**Comment:**			
**Sample weight:**	0.02054 g	**Sample Volume:**	0 cc		
**Outgas Time:**	24.0 hrs	**OutgasTemp:**	100.0 C		
**Analysis gas:**	Nitrogen	**Bath Temp:**	77.3 K		
**Press. Tolerance:**	0.070/0.070 (ads/des)	**Equil time:**	300/300 sec (ads/des)	**Equil timeout:**	600/600 sec (ads/des)
**Analysis Time:**	1049.2 min	**End of run:**	2017/11/16 2:39:01	**Instrument:**	Nova Station B
**Cell ID:**	1			**F/W version:**	0.00

Data Reduction Parameters

**t-Method**	**Calc. method:** de Boer				
**BJH/DH method**	**Moving pt. avg.:** 3	**Ignoring P-tags below 0.35 P/Po**			
**Adsorbate**	**Nitrogen**	**Temperature**	77.350K		
	**Molec. Wt.:** 28.013 g	**Cross Section:**	16.200 Ĺ	**Liquid Density:**	0.808 g/cc
	**Contact Angle:** 0.0 degrees	**Surf. Tension:**	8.850 erg/cm		

BJH method Desorption dV(log)

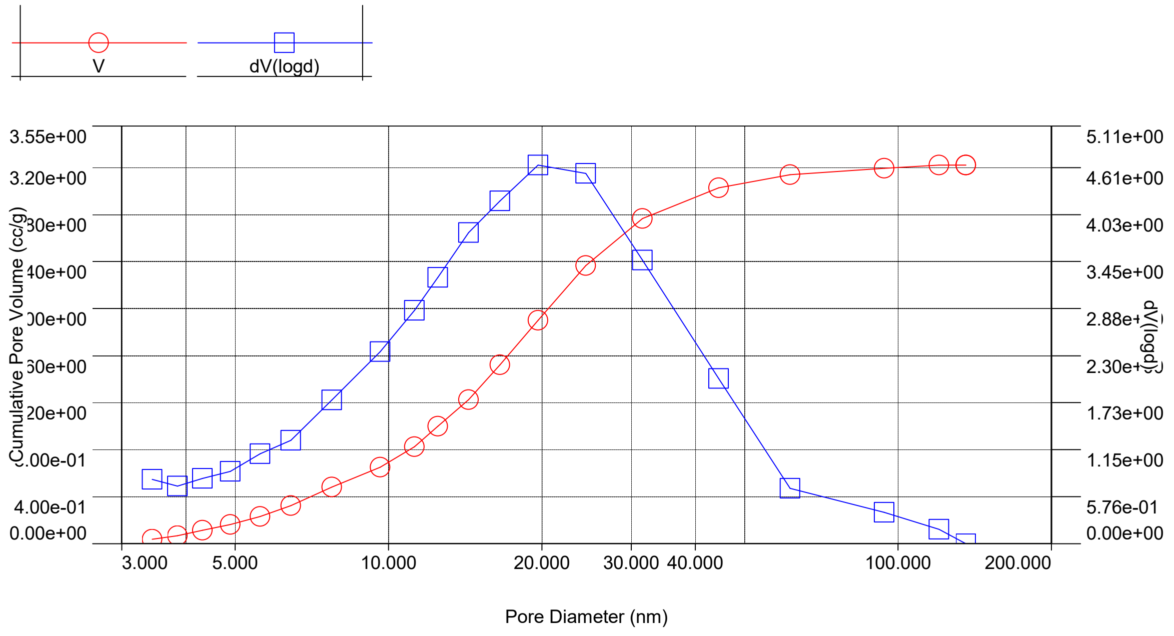

BJH desorption summary

**Surface Area =**	903.116 m,/g
**Pore Volume =**	3.223 cc/g
**Pore Diameter Dv(d) =**	14.341 nm

Analysis
Operator: AerogelLab **Date:**2017/11/16
Sample ID: P 77 I **Filename:**

Report
Operator: AerogelLab **Date:**9/24/2020
C:\Users\User\Documents\Munka\SEM cikk\Porozimetria\P 77 I 125 1.qps

BJH Pore Size Distribution Desorption

Diameter [nm]	Pore Volume [cc/g]	Pore Surf Area [m˛/g]	dV(d) [cc/nm/g]	dS(d) [m˛/nm/g]	dV(logd) [cc/g]	dS(logd) [cc/g]
3.4401	3.7534e-02	4.3643e+01	9.9530e-02	1.1573e+02	7.8759e-01	9.1578e+02
3.8567	7.0960e-02	7.7207e+01	8.0813e-02	8.5566e+01	7.0823e-01	7.4354e+02
4.3131	1.1537e-01	1.1715e+02	7.9223e-02	7.2773e+01	8.0146e-01	7.2868e+02
4.9002	1.6450e-01	1.5671e+02	7.7980e-02	6.3629e+01	8.8790e-01	7.1724e+02
5.5957	2.3307e-01	2.0528e+02	8.5309e-02	6.1450e+01	1.1034e+00	7.8438e+02
6.4348	3.2613e-01	2.5968e+02	8.2728e-02	5.0637e+01	1.2633e+00	7.6015e+02
7.7501	4.8165e-01	3.3510e+02	9.5160e-02	4.8496e+01	1.7628e+00	8.7352e+02
9.6346	6.4994e-01	4.0722e+02	1.0534e-01	4.4318e+01	2.3512e+00	9.6758e+02
11.2405	8.2396e-01	4.7191e+02	1.1192e-01	4.0719e+01	2.8601e+00	1.0288e+03
12.5182	9.9892e-01	5.2611e+02	1.1208e-01	3.5749e+01	3.2652e+00	1.0310e+03
14.3409	1.2260e+00	5.8806e+02	1.1362e-01	3.1595e+01	3.8072e+00	1.0448e+03
16.5700	1.5217e+00	6.5783e+02	1.0936e-01	2.6599e+01	4.1961e+00	1.0051e+03
19.6579	1.9018e+00	7.3230e+02	1.0212e-01	2.1170e+01	4.6379e+00	9.3787e+02
24.3581	2.3649e+00	8.0652e+02	8.1352e-02	1.3834e+01	4.5358e+00	7.4595e+02
31.4921	2.7666e+00	8.6141e+02	5.3489e-02	7.7710e+00	3.4731e+00	4.8986e+02
44.5107	3.0281e+00	8.9091e+02	2.5232e-02	2.9853e+00	2.0192e+00	2.3070e+02
61.3639	3.1401e+00	8.9906e+02	5.5111e-03	4.3179e-01	6.7867e-01	5.0152e+01
93.7899	3.1931e+00	9.0186e+02	2.2463e-03	1.2697e-01	3.8388e-01	2.0488e+01
120.4924	3.2221e+00	9.0308e+02	7.7881e-04	3.1308e-02	1.7777e-01	7.0525e+00
136.0894	3.2231e+00	9.0312e+02	0.0000e+00	0.0000e+00	0.0000e+00	0.0000e+00

Silica-casein aerogel

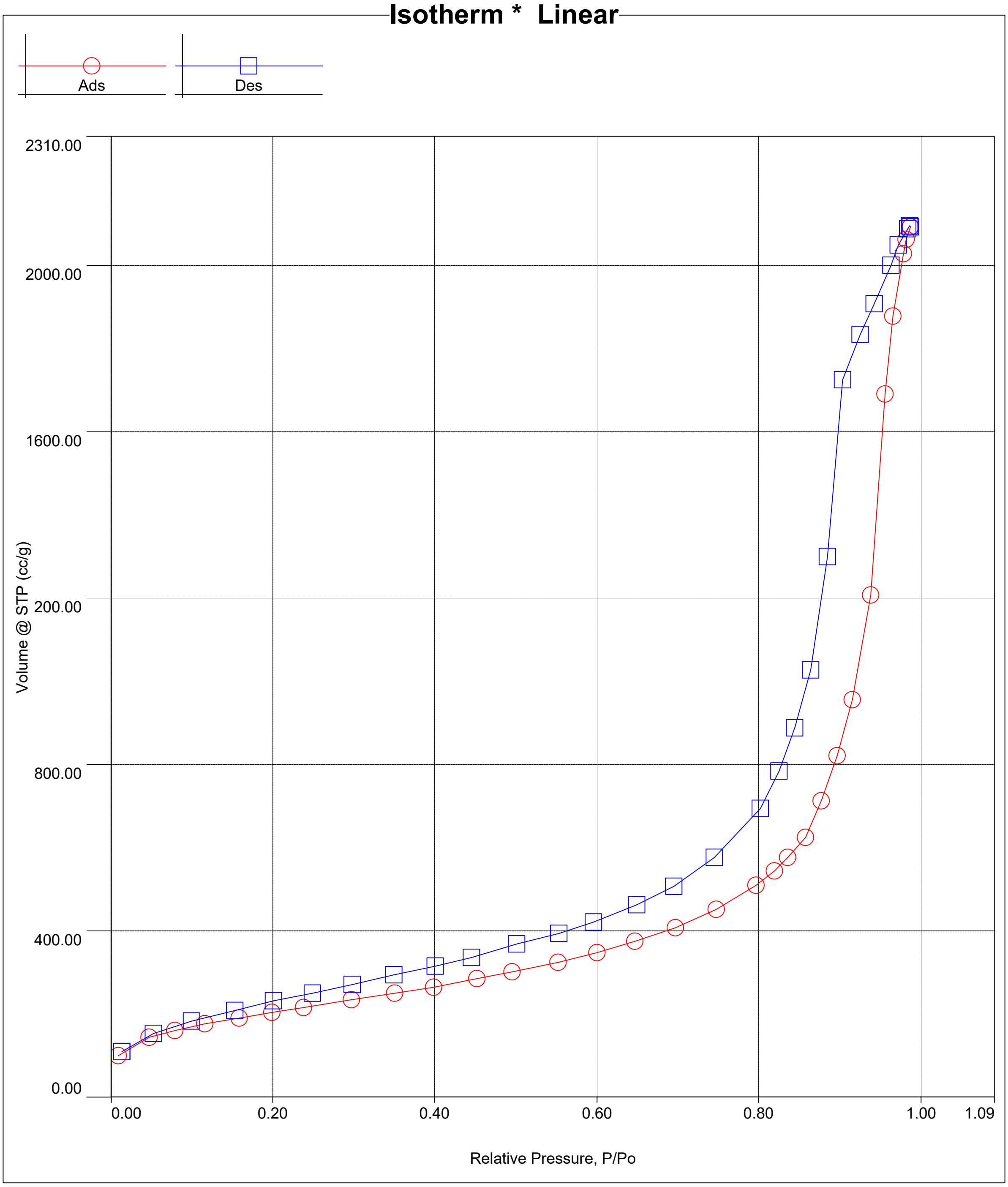

Quantachrome NovaWin - Data Acquisition and Reduction
for NOVA instruments
©1994-2010, Quantachrome Instruments
version 11.0

Quantachrome INSTRUMENTS *Optimizing particle performance*

**Analysis**		**Report**	
**Operator:**AerogelLab	**Date:**2018/01/27	**Operator:**AerogelLab	**Date:**9/24/2020
**Sample ID:** HA4	**Filename:**	C:\Users\User\Documents\Munka\SEM cikk\Porozimetria\HA4.qps	

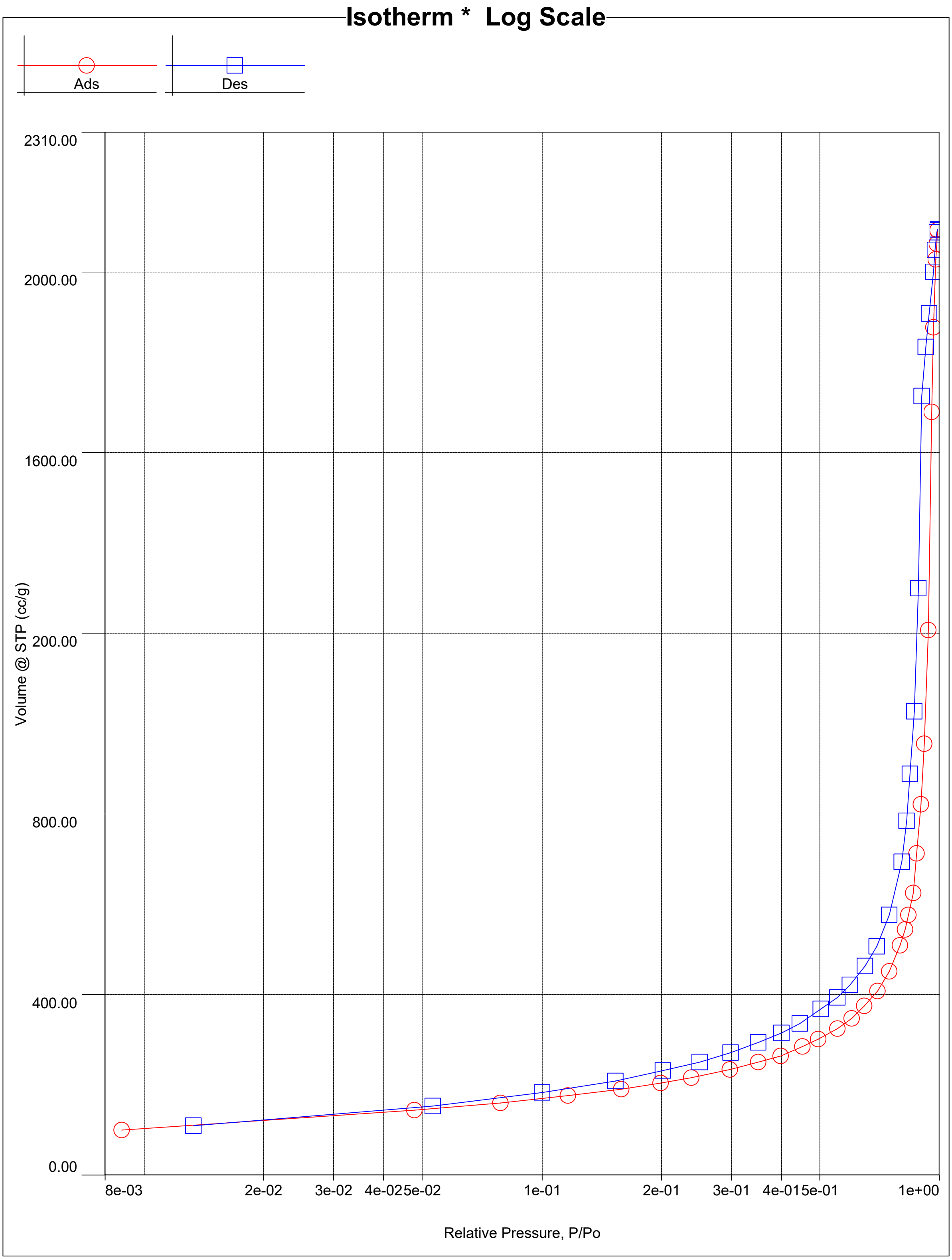

Analysis
Operator: AerogelLab **Date:**2018/01/27
Sample ID: HA4 **Filename:**

Report
Operator: AerogelLab **Date:**9/24/2020
C:\Users\User\Documents\Munka\SEM cikk\Porozimetria\HA4.qps

Isotherm

Relative Pressure	Volume @ STP [cc/g]	Relative Pressure	Volume @ STP [cc/g]	Relative Pressure	Volume @ STP [cc/g]
8.77500e-03	101.1404	8.56941e-01	625.1418	8.24574e-01	784.1856
4.78270e-02	143.7760	8.77123e-01	713.3493	8.01400e-01	693.5259
7.87160e-02	160.2262	8.96353e-01	820.1728	7.45418e-01	576.6899
1.16419e-01	175.9236	9.15425e-01	954.6318	6.94686e-01	507.7773
1.58160e-01	190.7017	9.37453e-01	1207.8373	6.49347e-01	463.4785
1.99166e-01	203.9440	9.55767e-01	1689.8578	5.95204e-01	422.0319
2.38332e-01	216.0921	9.65638e-01	1876.5775	5.52965e-01	394.9907
2.97407e-01	233.8805	9.77795e-01	2026.8673	5.01353e-01	367.7287
3.50058e-01	249.6052	9.81350e-01	2062.0139	4.44745e-01	336.4986
3.98223e-01	265.2384	9.87125e-01	2092.7407	4.00220e-01	315.6704
4.51508e-01	285.6075	9.85964e-01	2093.4517	3.49712e-01	294.5530
4.95946e-01	302.2677	9.83325e-01	2086.3149	2.98359e-01	271.7861
5.52288e-01	324.8879	9.72156e-01	2047.0381	2.49256e-01	251.7095
6.00157e-01	348.1961	9.63021e-01	2000.1331	2.01552e-01	231.6188
6.46953e-01	374.6130	9.42175e-01	1907.2833	1.53084e-01	209.3292
6.96986e-01	407.4262	9.24842e-01	1832.8511	1.00109e-01	183.0786
7.47177e-01	450.4799	9.02821e-01	1724.0587	5.31840e-02	153.4948
7.95740e-01	509.0696	8.84319e-01	1299.3405	1.33250e-02	110.3501
8.18611e-01	543.3544	8.64116e-01	1027.2841		
8.35692e-01	576.7286	8.44388e-01	887.7612		

Quantachrome INSTRUMENTS *Optimizing particle performance*

**Analysis**				**Report**		
**Operator:** AerogelLab		**Date:** 2018/01/27		**Operator:** AerogelLab	**Date:** 9/24/2020	
**Sample ID:** HA4		**Filename:**	C:\Users\User\Documents\Munka\SEM cikk\Porozimetria\HA4.qps			
**Sample Desc:**	kazeines	**Comment:**				
**Sample weight:**	0.02498 g	**Sample Volume:**	0 cc			
**Outgas Time:**	22.0 hrs	**OutgasTemp:**	50.0 C			
**Analysis gas:**	Nitrogen	**Bath Temp:**	77.3 K			
**Press. Tolerance:**	0.070/0.070 (ads/des)	**Equil time:**	300/300 sec (ads/des)	**Equil timeout:**	600/600 sec (ads/des)	
**Analysis Time:**	1097.4 min	**End of run:**	2018/01/27 9:53:21	**Instrument:**	Nova Station B	
**Cell ID:**	1			**F/W version:**	0.00	

Data Reduction Parameters

Adsorbate **Nitrogen** **Temperature** 77.350K
Molec. Wt.: 28.013 g **Cross Section:** 16.200 Ĺ **Liquid Density:** 0.808 g/cc

MBET summary

Slope = 4.578
Intercept = 6.565e-02
Correlation coefficient, r = 0.999986
C constant= 70.737

Surface Area = 749.963 m./g

Multi-Point BET Plot

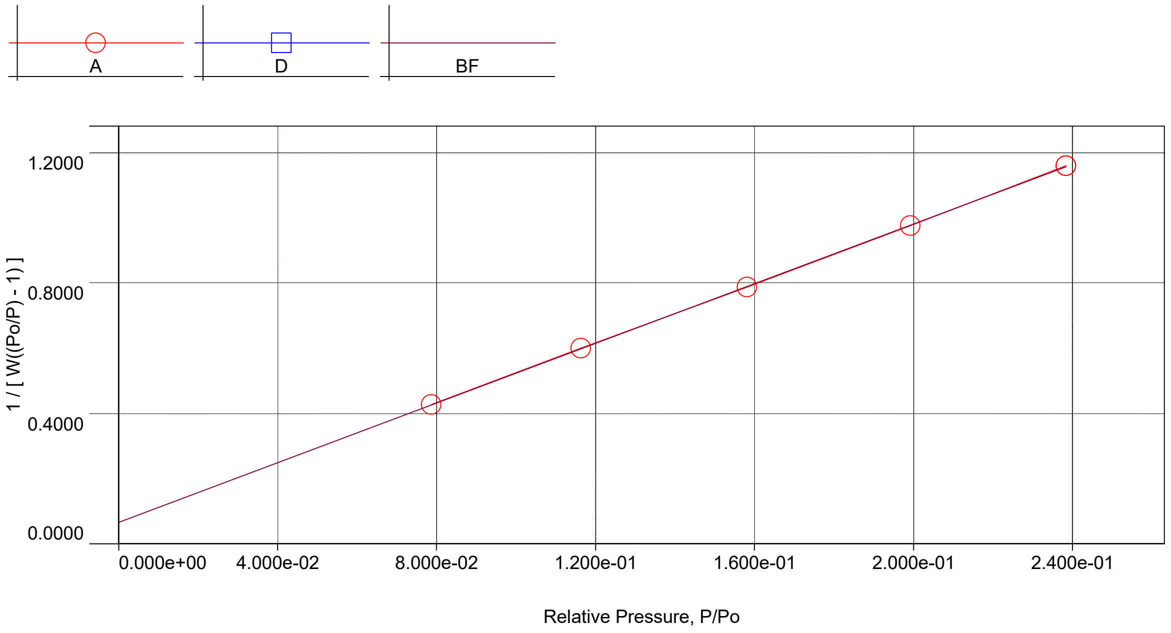

Multi-Point BET

Relative Pressure [P/Po]	Volume @ STP [cc/g]	1 / [W((Po/P) - 1)]
7.87160e-02	160.2262	4.2666e-01
1.16419e-01	175.9236	5.9924e-01
1.58160e-01	190.7017	7.8825e-01
1.99166e-01	203.9440	9.7569e-01
2.38332e-01	216.0921	1.1586e+00

Quantachrome NovaWin - Data Acquisition and Reduction
for NOVA instruments
©1994-2010, Quantachrome Instruments
version 11.0

Quantachrome INSTRUMENTS
Optimizing particle performance

**Analysis**				**Report**			
**Operator:**AerogelLab			**Date:**2018/01/27	**Operator:**AerogelLab		**Date:**9/24/2020	
**Sample ID:** HA4		**Filename:**		C:\Users\User\Documents\Munka\SEM cikk\Porozimetria\HA4.qps			
**Sample Desc:**	kazeines	**Comment:**					
**Sample weight:**	0.02498 g	**Sample Volume:**		0 cc			
**Outgas Time:**	22.0 hrs	**OutgasTemp:**		50.0 C			
**Analysis gas:**	Nitrogen	**Bath Temp:**		77.3 K			
**Press. Tolerance:**	0.070/0.070 (ads/des)	**Equil time:**		300/300 sec (ads/des)	**Equil timeout:**	600/600 sec (ads/des)	
**Analysis Time:**	1097.4 min	**End of run:**		2018/01/27 9:53:21	**Instrument:**	Nova Station B	
**Cell ID:**	1				**F/W version:**	0.00	

Data Reduction Parameters

**t-Method**	**Calc. method:** de Boer				
**BJH/DH method**	**Moving pt. avg.:** 3	**Ignoring P-tags below 0.35 P/Po**			
**Adsorbate**	**Nitrogen**	**Temperature**	77.350K		
	**Molec. Wt.:** 28.013 g	**Cross Section:**	16.200 Å^2	**Liquid Density:**	0.808 g/cc
	**Contact Angle:** 0.0 degrees	**Surf. Tension:**	8.850 erg/cm^2		

BJH method Desorption dV(log)

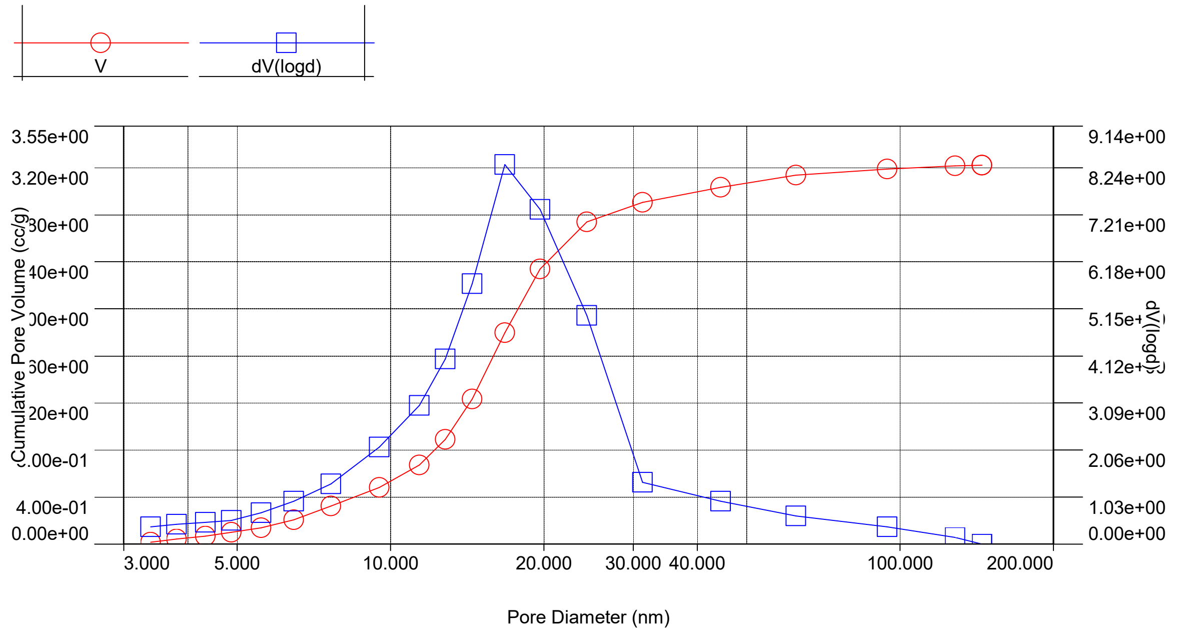

BJH desorption summary

**Surface Area =**	874.019 m^2/g
**Pore Volume =**	3.220 cc/g
**Pore Diameter Dv(d) =**	16.759 nm

Analysis
Operator: AerogelLab **Date:**2018/01/27
Sample ID: HA4 **Filename:**

Report
Operator: AerogelLab **Date:**9/24/2020
C:\Users\User\Documents\Munka\SEM cikk\Porozimetria\HA4.qps

BJH Pore Size Distribution Desorption

Diameter [nm]	Pore Volume [cc/g]	Pore Surf Area [m,/g]	dV(d) [cc/nm/g]	dS(d) [m,/nm/g]	dV(logd) [cc/g]	dS(logd) [cc/g]
3.3910	1.6223e-02	1.9137e+01	4.8141e-02	5.6786e+01	3.7558e-01	4.4303e+02
3.8091	4.4204e-02	4.7702e+01	4.9364e-02	5.2426e+01	4.3114e-01	4.5411e+02
4.3329	6.9873e-02	7.1895e+01	4.8986e-02	4.6348e+01	4.8173e-01	4.5061e+02
4.8771	9.9528e-02	9.5359e+01	4.4525e-02	3.5963e+01	5.1216e-01	4.0949e+02
5.5781	1.4118e-01	1.2426e+02	5.2485e-02	3.7291e+01	6.8847e-01	4.8263e+02
6.4770	2.0820e-01	1.6392e+02	6.0792e-02	3.6976e+01	9.3385e-01	5.5866e+02
7.6505	3.2542e-01	2.2027e+02	7.1461e-02	3.6370e+01	1.3208e+00	6.5595e+02
9.5248	4.8195e-01	2.8553e+02	9.3608e-02	3.8909e+01	2.1230e+00	8.5977e+02
11.3835	6.7222e-01	3.5418e+02	1.1494e-01	4.0512e+01	3.0377e+00	1.0564e+03
12.8191	8.8977e-01	4.2098e+02	1.3573e-01	4.2230e+01	4.0526e+00	1.2486e+03
14.5140	1.2338e+00	5.1146e+02	1.6503e-01	4.4506e+01	5.6967e+00	1.5176e+03
16.7586	1.7965e+00	6.3898e+02	2.0683e-01	4.8058e+01	8.3023e+00	1.9011e+03
19.6964	2.3362e+00	7.5247e+02	1.6987e-01	3.6581e+01	7.3285e+00	1.5608e+03
24.3171	2.7373e+00	8.2784e+02	1.0459e-01	2.0354e+01	5.0077e+00	9.6064e+02
31.1958	2.9070e+00	8.5001e+02	2.0383e-02	2.9236e+00	1.3592e+00	1.8651e+02
44.5384	3.0344e+00	8.6251e+02	1.0337e-02	1.1128e+00	9.3712e-01	9.4430e+01
62.5190	3.1379e+00	8.7008e+02	4.9490e-03	3.8139e-01	6.1877e-01	4.4997e+01
94.1138	3.1906e+00	8.7285e+02	2.2295e-03	1.2656e-01	3.7899e-01	2.0351e+01
127.9554	3.2163e+00	8.7390e+02	6.5409e-04	2.5767e-02	1.5362e-01	5.9318e+00
144.8833	3.2202e+00	8.7402e+02	0.0000e+00	0.0000e+00	0.0000e+00	0.0000e+00

Ca-alginate aerogel

Isotherm

Relative Pressure	Volume @ STP [cc/g]	Relative Pressure	Volume @ STP [cc/g]	Relative Pressure	Volume @ STP [cc/g]
2.59200e-02	102.6731	7.54760e-01	294.1950	8.93190e-01	550.4418
5.82430e-02	120.1886	8.12890e-01	326.4201	8.62659e-01	437.4172
8.71830e-02	129.5685	8.49420e-01	354.7062	8.32531e-01	374.6402
1.36881e-01	141.5530	8.86453e-01	393.6490	7.97703e-01	337.7549
1.56335e-01	145.6977	9.45835e-01	614.2334	7.43107e-01	306.8555
1.77167e-01	149.6675	9.64484e-01	1136.5291	6.40881e-01	265.1518
2.14866e-01	156.9812	9.70365e-01	1643.3116	5.53176e-01	238.4861
2.72661e-01	167.7071	9.84136e-01	4277.4209	4.51679e-01	211.4411
3.18186e-01	176.2322	9.86395e-01	4869.3389	3.49126e-01	188.3932
3.64298e-01	185.0471	9.80568e-01	4833.2012	2.45140e-01	166.1525
4.60695e-01	204.5588	9.73511e-01	4756.2344	1.36932e-01	141.9993
5.65770e-01	229.9326	9.58024e-01	3911.9836		
6.66993e-01	259.4377	9.42427e-01	2076.4856		

**Analysis**			**Report**			
**Operator:**	Kammlott	**Date:**2001/02/22	**Operator:**	quantachrome	**Date:**2019-03-20	
**Sample ID:**	11.03.2019	**Filename:**	Pavel_20190311_Kalmar_alginate.qps			

Isotherm : Linear

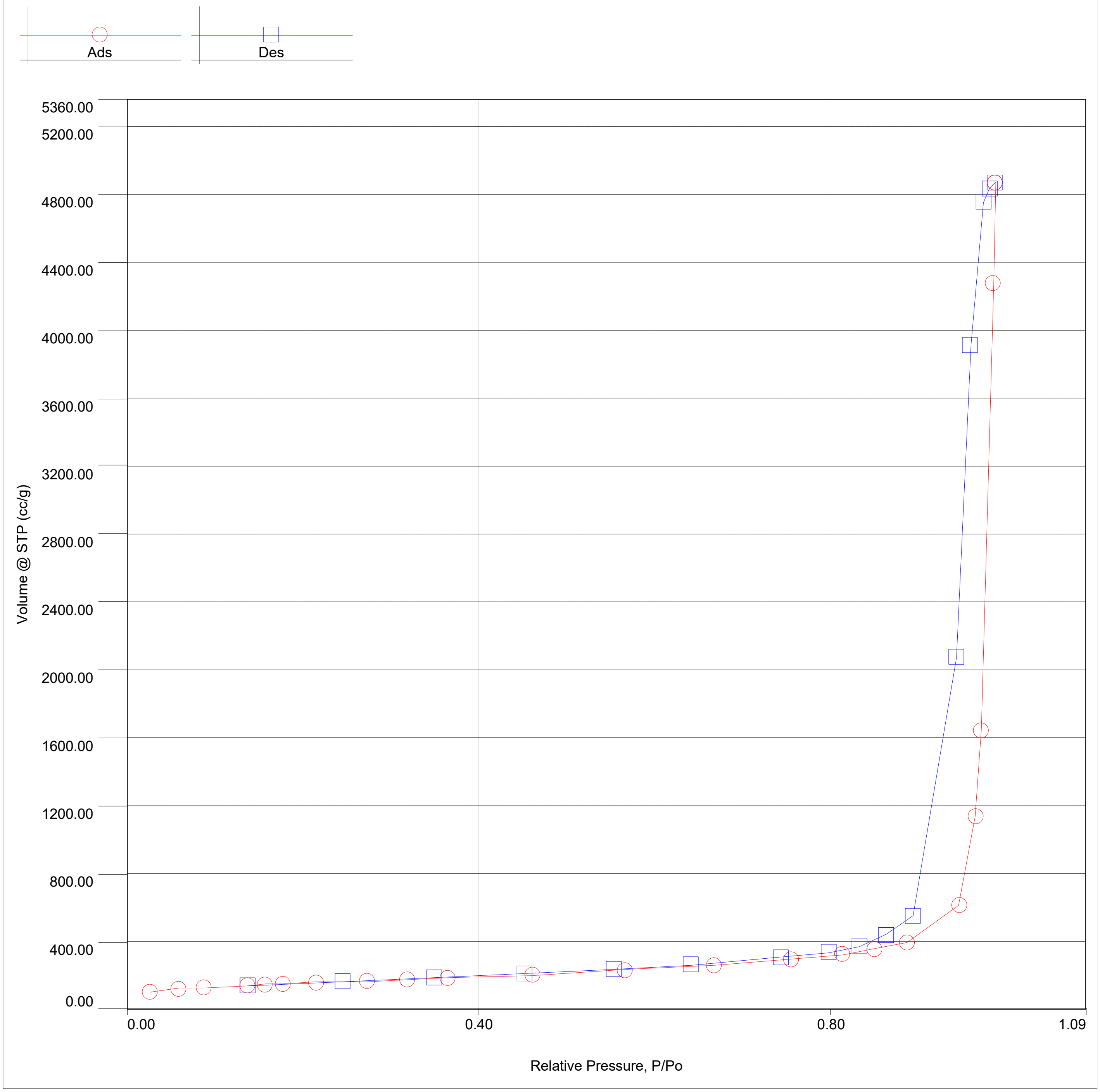

**Analysis**			**Report**		
**Operator:**	Kammlott	**Date:**2001/02/22	**Operator:**	quantachrome	**Date:**2019-03-20
**Sample ID:**	11.03.2019	**Filename:**	Pavel_20190311_Kalmar_alginate.qps		

BJH method Desorption dV(log)

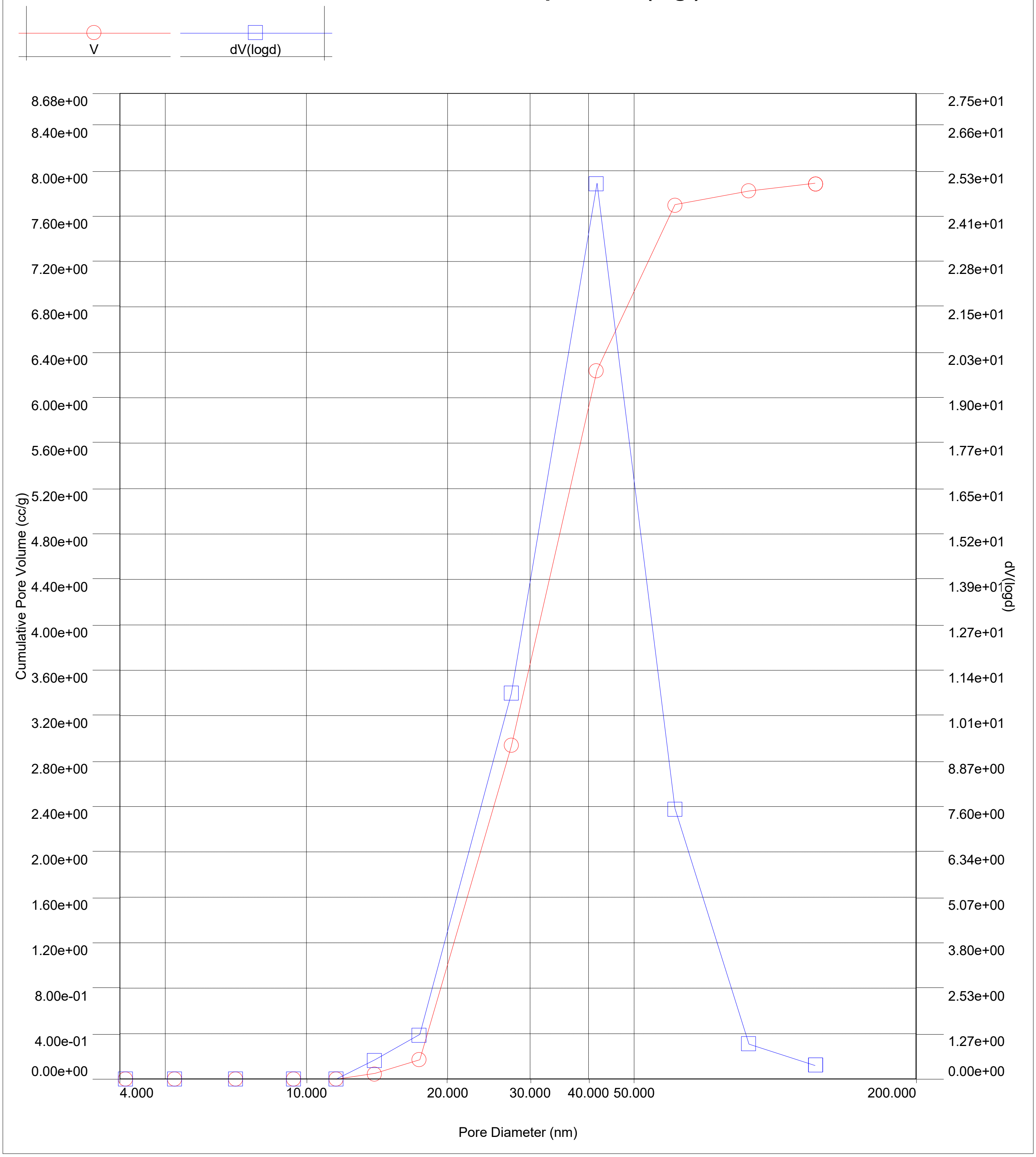

**Analysis**			**Report**		
**Operator:**	Kammlott	**Date:**2001/02/22	**Operator:**	quantachrome	**Date:**2019-03-20
**Sample ID:**	11.03.2019	**Filename:**	Pavel_20190311_Kalmar_alginate.qps		

Volume/Area summary

Surface Area Data

MultiPoint BET	5.444e+02 m²/g
BJH method cumulative desorption surface area	8.672e+02 m²/g
DH method cumulative desorption surface area	8.769e+02 m²/g
DFT cumulative surface area	5.368e+02 m²/g

Pore Volume Data

Total pore volume for pores with Diameter less than 143.06 nm at P/Po = 0.986395	7.532e+00 cc/g
BJH method cumulative desorption pore volume	7.882e+00 cc/g
DH method cumulative desorption pore volume	7.660e+00 cc/g
HK method micropore volume	2.240e-01 cc/g
SF method micropore volume	1.553e-01 cc/g
DFT method cumulative pore volume	6.075e+00 cc/g

Pore Size Data

Average pore Diameter	5.534e+01 nm
BJH method desorption pore Diameter (Mode Dv(d))	4.150e+01 nm
DH method desorption pore Diameter (Mode Dv(d))	4.150e+01 nm
HK method pore Diameter (Mode)	3.675e-01 nm
SF method pore Diameter (Mode)	4.523e-01 nm
DFT pore Diameter (Mode)	2.875e+01 nm

Quantachrome INSTRUMENTS Optimizing particle performance

Polyimide aerogel

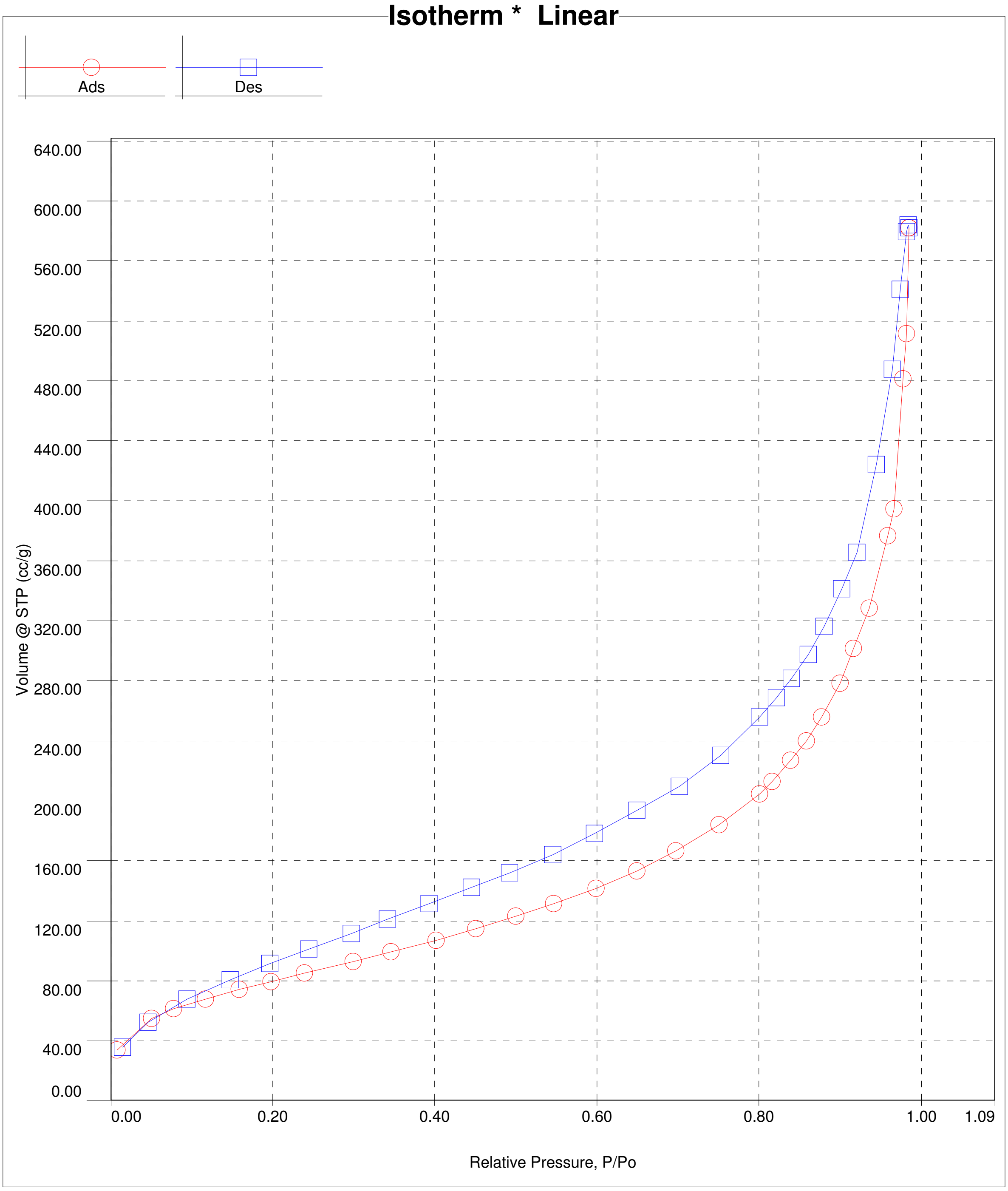

Quantachrome NovaWin - Data Acquisition and Reduction
for NOVA instruments
©1994-2010, Quantachrome Instruments
version 11.0

Quantachrome INSTRUMENTS
Optimizing particle performance

Analysis
Operator: AerogelLab **Date:**2019/07/19
Sample ID: MK_PI1 **Filename:**

Report
Operator: AerogelLab **Date:**7/22/2019
C:\QCdata\Physisorb\MK_PI1.qps

Isotherm * Log Scale

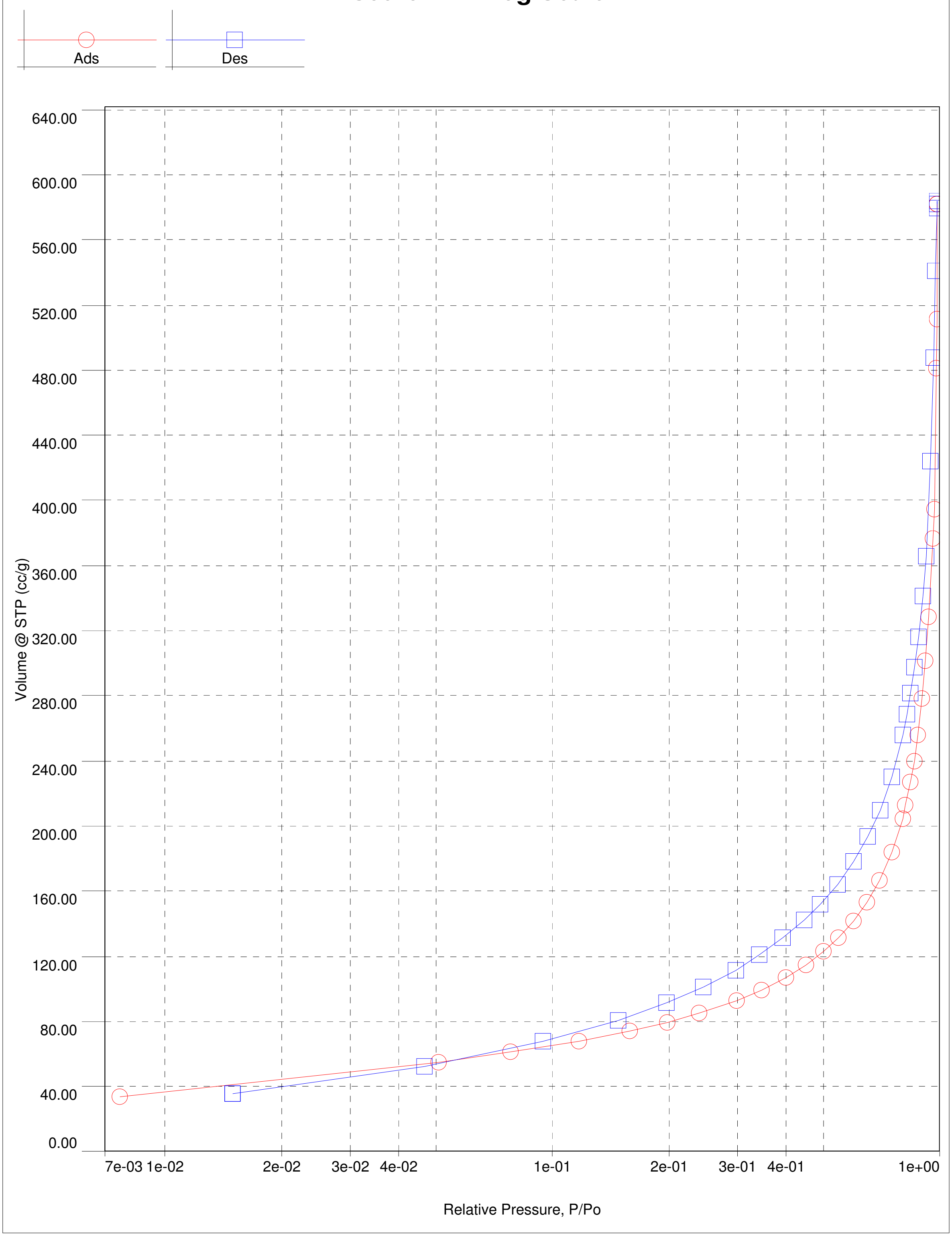

Quantachrome INSTRUMENTS Optimizing particle performance

Analysis
Operator: AerogelLab **Date:**2019/07/19
Sample ID: MK_PI1 **Filename:**

Report
Operator: AerogelLab **Date:**7/22/2019
C:\QCdata\Physisorb\MK_PI1.qps

Isotherm

Relative Pressure	Volume @ STP [cc/g]	Relative Pressure	Volume @ STP [cc/g]	Relative Pressure	Volume @ STP [cc/g]
7.64500e-03	33.8427	8.58326e-01	239.9075	8.21274e-01	268.3664
5.08940e-02	55.1893	8.76685e-01	256.0341	8.00427e-01	255.6707
7.79090e-02	61.0593	8.99509e-01	278.6115	7.52719e-01	230.1391
1.17201e-01	67.9514	9.15824e-01	301.3740	7.00930e-01	209.8923
1.58378e-01	73.9328	9.35551e-01	328.1953	6.49491e-01	193.7476
1.98136e-01	79.3697	9.58892e-01	376.6359	5.97164e-01	178.0457
2.38803e-01	84.8482	9.66144e-01	394.3131	5.45659e-01	164.1608
2.98731e-01	92.6982	9.77644e-01	481.1165	4.91760e-01	152.0835
3.45937e-01	99.2990	9.81731e-01	511.6534	4.45500e-01	141.8961
4.01268e-01	107.0735	9.85223e-01	581.6766	3.92904e-01	131.4653
4.50235e-01	114.5202	9.84204e-01	583.6115	3.41449e-01	120.9214
4.99805e-01	122.6853	9.82044e-01	579.5242	2.97429e-01	111.4797
5.46131e-01	131.2147	9.73629e-01	540.7205	2.44658e-01	101.0841
5.98640e-01	141.7751	9.63656e-01	487.8535	1.96521e-01	91.4068
6.48829e-01	153.2542	9.44177e-01	423.9698	1.47591e-01	80.5803
6.97337e-01	166.4051	9.20603e-01	365.7083	9.45220e-02	67.5037
7.50951e-01	183.8830	9.01815e-01	341.2431	4.67700e-02	52.5707
8.00325e-01	204.3131	8.80496e-01	316.0504	1.49470e-02	35.4238
8.15513e-01	213.0392	8.60515e-01	297.3838		
8.38425e-01	226.6642	8.40265e-01	281.5996		

Quantachrome NovaWin - Data Acquisition and Reduction
for NOVA instruments
©1994-2010, Quantachrome Instruments
version 11.0

Quantachrome INSTRUMENTS
Optimizing particle performance

**Analysis**			**Report**		
**Operator:** AerogelLab	**Date:** 2019/07/19		**Operator:** AerogelLab	**Date:** 7/22/2019	
**Sample ID:** MK_PI1	**Filename:**		C:\QCdata\Physisorb\MK_PI1.qps		
**Sample Desc:**	**Comment:**				
**Sample weight:**	0.0285 g	**Sample Volume:**	0 cc		
**Outgas Time:**	24.0 hrs	**OutgasTemp:**	80.0 C		
**Analysis gas:**	Nitrogen	**Bath Temp:**	77.3 K		
**Press. Tolerance:**	0.070/0.070 (ads/des)	**Equil time:**	300/300 sec (ads/des)	**Equil timeout:**	600/600 sec (ads/des)
**Analysis Time:**	726.7 min	**End of run:**	2019/07/19 4:20:48	**Instrument:**	Nova Station B
**Cell ID:**	1			**F/W version:**	0.00

Data Reduction Parameters

**Adsorbate**	**Nitrogen**	**Temperature**	77.350K		
	**Molec. Wt.:** 28.013 g	**Cross Section:**	16.200 Å²	**Liquid Density:**	0.808 g/cc

MBET summary

Slope = 11.497
Intercept = 2.138e-01
Correlation coefficient, r = 0.999997
C constant= 54.784

Surface Area = 297.383 m²/g

Multi-Point BET Plot

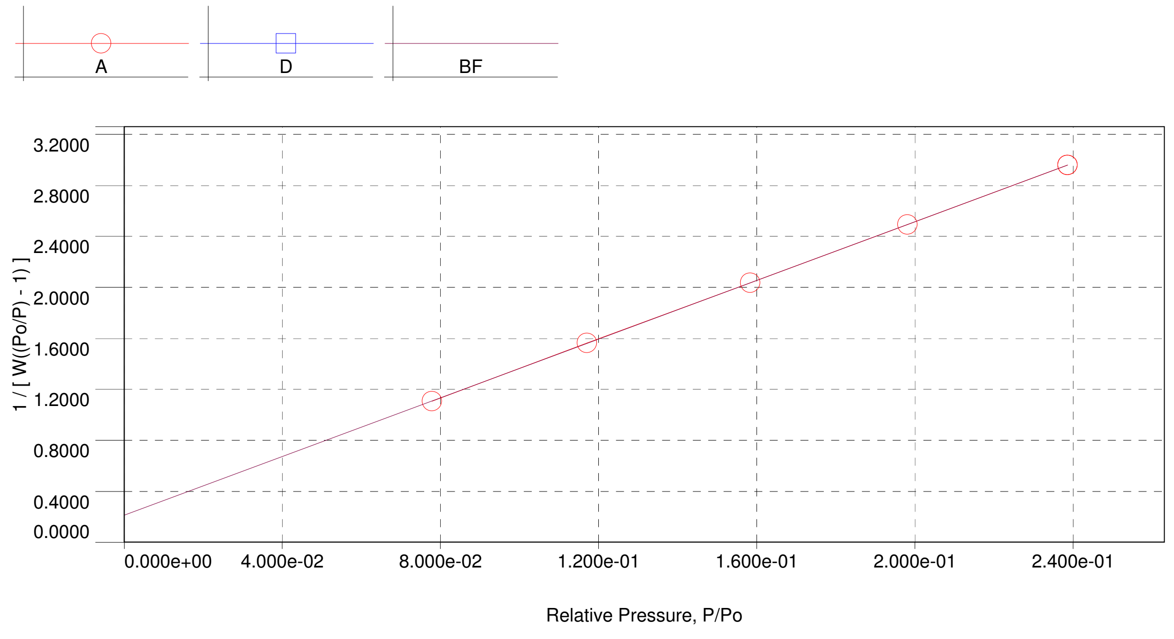

Multi-Point BET

Relative Pressure [P/Po]	Volume @ STP [cc/g]	1 / [W((Po/P) - 1)]	Relative Pressure [P/Po]	Volume @ STP [cc/g]	1 / [W((Po/P) - 1)]
7.79090e-02	61.0593	1.1072e+00	1.98136e-01	79.3697	2.4909e+00
1.17201e-01	67.9514	1.5632e+00	2.38803e-01	84.8482	2.9584e+00
1.58378e-01	73.9328	2.0365e+00			

Quantachrome INSTRUMENTS Optimizing particle performance

Analysis
Operator: AerogelLab **Date:** 2019/07/19
Sample ID: MK_PI1 **Filename:** C:\QCdata\Physisorb\MK_PI1.qps
Sample Desc: **Comment:**
Sample weight: 0.0285 g **Sample Volume:** 0 cc
Outgas Time: 24.0 hrs **OutgasTemp:** 80.0 C
Analysis gas: Nitrogen **Bath Temp:** 77.3 K
Press. Tolerance: 0.070/0.070 (ads/des) **Equil time:** 300/300 sec (ads/des) **Equil timeout:** 600/600 sec (ads/des)
Analysis Time: 726.7 min **End of run:** 2019/07/19 4:20:48 **Instrument:** Nova Station B
Cell ID: 1 **F/W version:** 0.00

Report
Operator: AerogelLab **Date:** 7/22/2019

Data Reduction Parameters

t-Method **Calc. method:** de Boer
BJH/DH method **Moving pt. avg.:** off **Ignoring P-tags below 0.35 P/Po**
Adsorbate **Nitrogen** **Temperature** 77.350K
Molec. Wt.: 28.013 g **Cross Section:** 16.200 Å² **Liquid Density:** 0.808 g/cc
Contact Angle: 0.0 degrees **Surf. Tension:** 8.850 erg/cm²

BJH method Desorption dV(log)

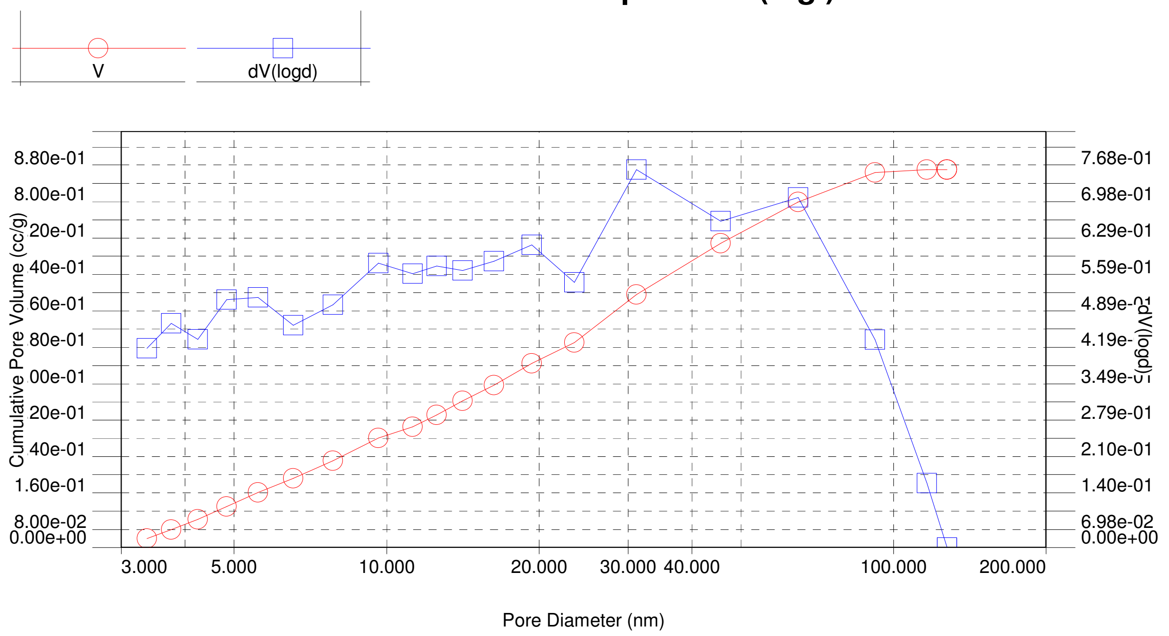

BJH desorption summary

Surface Area = 254.393 m²/g
Pore Volume = 0.831 cc/g
Pore Diameter Dv(d) = 3.767 nm

Analysis
Operator: AerogelLab **Date:**2019/07/19
Sample ID: MK_PI1 **Filename:**

Report
Operator: AerogelLab **Date:**7/22/2019
C:\QCdata\Physisorb\MK_PI1.qps

BJH Pore Size Distribution Desorption

Diameter [nm]	Pore Volume [cc/g]	Pore Surf Area [m²/g]	dV(d) [cc/nm/g]	dS(d) [m²/nm/g]	dV(logd) [cc/g]	dS(logd) [cc/g]
3.3683	1.9449e-02	2.3097e+01	4.9280e-02	5.8523e+01	3.8177e-01	4.5337e+02
3.7665	3.9382e-02	4.4265e+01	4.9601e-02	5.2676e+01	4.2977e-01	4.5641e+02
4.2452	6.2127e-02	6.5696e+01	4.0952e-02	3.8587e+01	3.9973e-01	3.7664e+02
4.8487	8.9886e-02	8.8597e+01	4.2595e-02	3.5139e+01	4.7484e-01	3.9172e+02
5.5931	1.2115e-01	1.1096e+02	3.7351e-02	2.6712e+01	4.8013e-01	3.4337e+02
6.5530	1.5184e-01	1.2969e+02	2.8342e-02	1.7300e+01	4.2668e-01	2.6045e+02
7.8491	1.9085e-01	1.4957e+02	2.5843e-02	1.3170e+01	4.6562e-01	2.3728e+02
9.6230	2.4120e-01	1.7050e+02	2.4702e-02	1.0268e+01	5.4529e-01	2.2666e+02
11.2491	2.6587e-01	1.7927e+02	2.0321e-02	7.2259e+00	5.2585e-01	1.8698e+02
12.5406	2.9150e-01	1.8745e+02	1.8725e-02	5.9724e+00	5.4015e-01	1.7229e+02
14.1516	3.2173e-01	1.9599e+02	1.6314e-02	4.6113e+00	5.3085e-01	1.5005e+02
16.2883	3.5726e-01	2.0471e+02	1.4678e-02	3.6044e+00	5.4947e-01	1.3494e+02
19.3140	4.0480e-01	2.1456e+02	1.3093e-02	2.7115e+00	5.8054e-01	1.2023e+02
23.5135	4.4976e-01	2.2221e+02	9.4311e-03	1.6044e+00	5.0886e-01	8.6565e+01
31.1008	5.5615e-01	2.3589e+02	1.0223e-02	1.3148e+00	7.2519e-01	9.3270e+01
45.5979	6.6856e-01	2.4575e+02	6.0478e-03	5.3054e-01	6.2609e-01	5.4923e+01
64.9015	7.5920e-01	2.5134e+02	4.5274e-03	2.7903e-01	6.7118e-01	4.1366e+01
91.9456	8.2405e-01	2.5416e+02	1.9036e-03	8.2813e-02	3.9836e-01	1.7330e+01
116.2680	8.3079e-01	2.5439e+02	4.6236e-04	1.5907e-02	1.2362e-01	4.2530e+00
127.7319	8.3079e-01	2.5439e+02	0.0000e+00	0.0000e+00	0.0000e+00	0.0000e+00

Polyamide aerogel

Isotherm * Linear

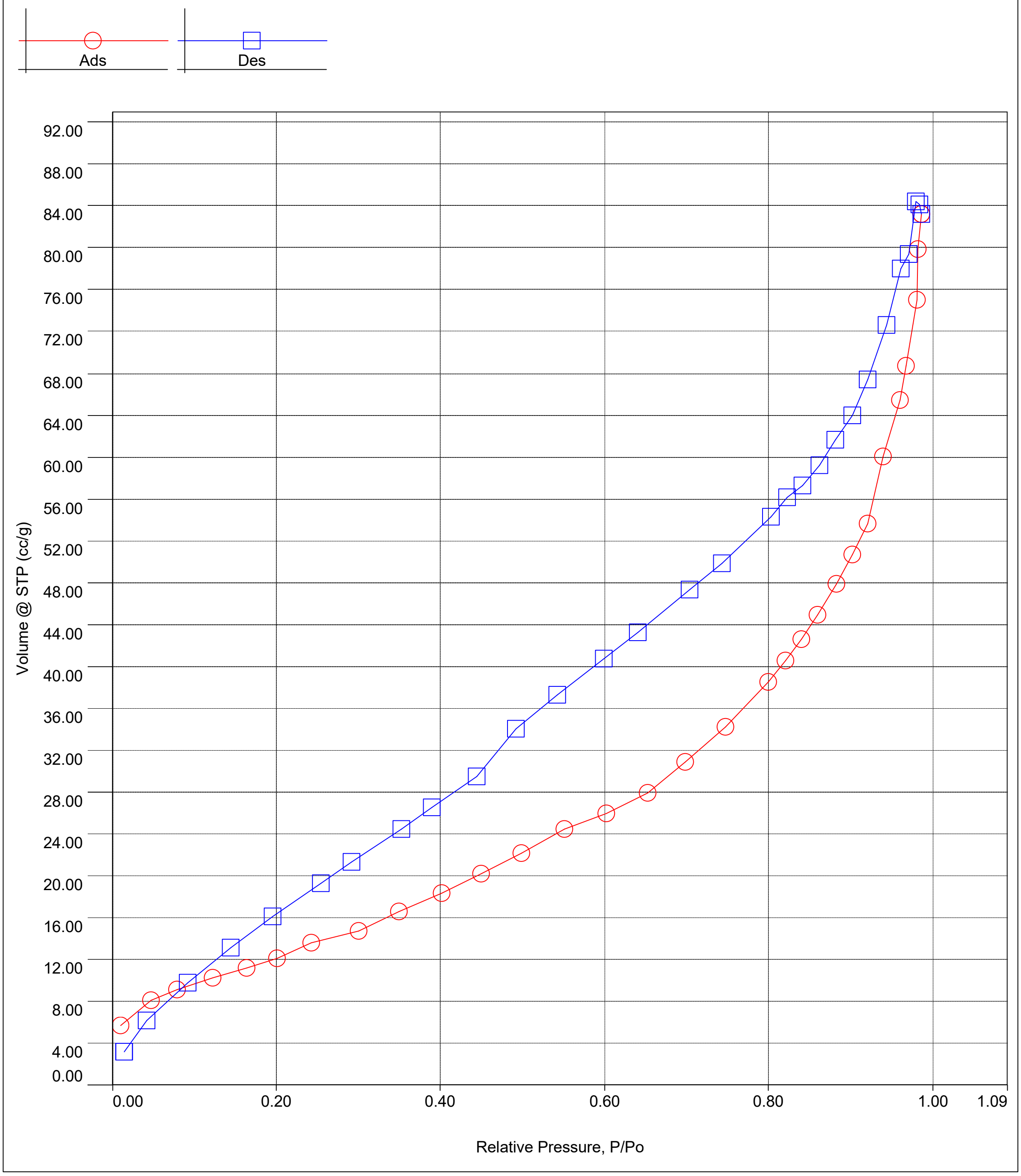

Quantachrome INSTRUMENTS
Optimizing particle performance

Analysis
Operator: AerogelLab **Date:**2019/09/05
Sample ID: MK_PA2 **Filename:**

Report
Operator: AerogelLab **Date:**4/21/2020
C:\Users\User\Documents\Munka\Polymer Aerogel\Poliamid aerogel\PA2014\Poroz

Isotherm * Log Scale

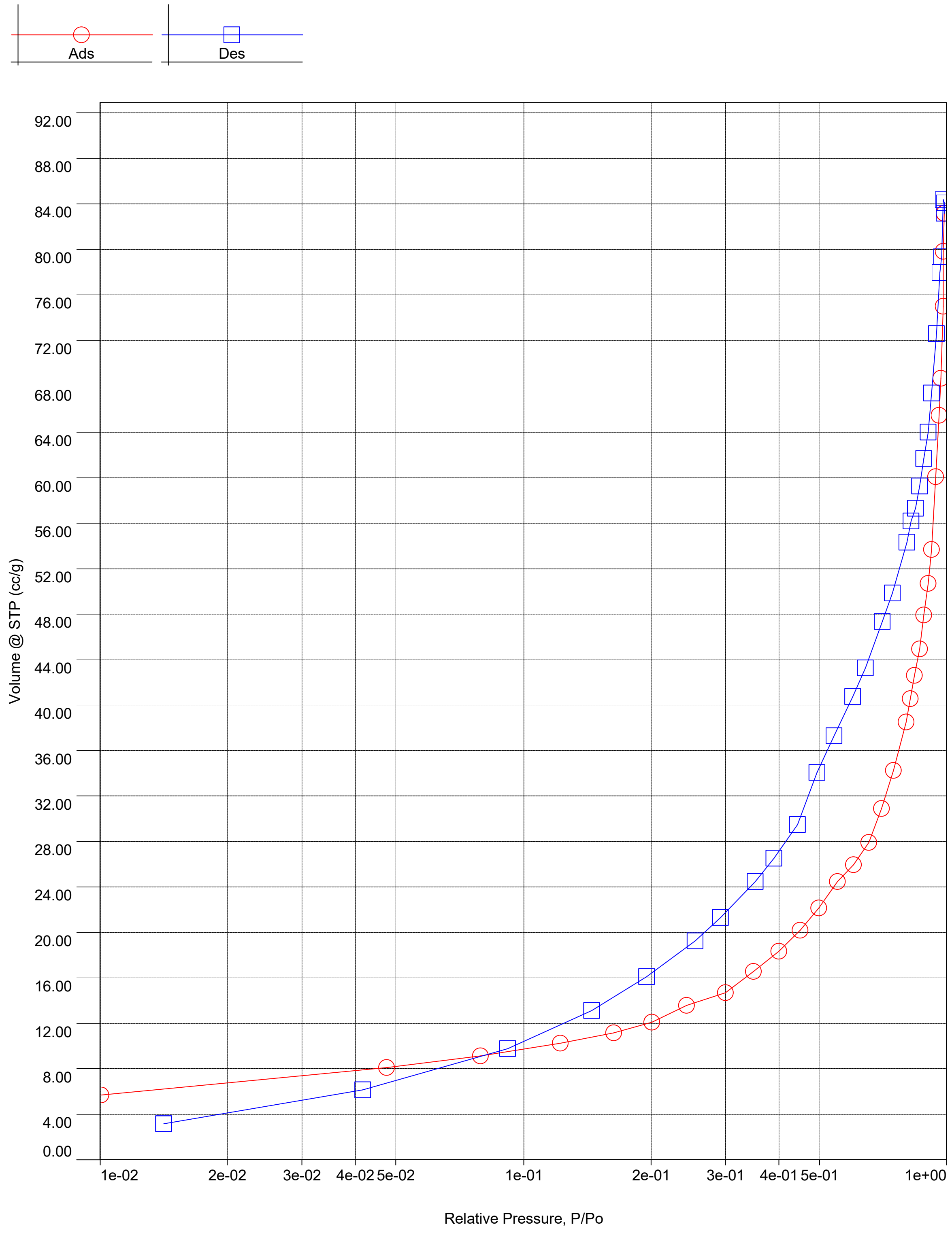

Analysis
Operator: AerogelLab **Date:**2019/09/05
Sample ID: MK_PA2 **Filename:**

Report
Operator: AerogelLab **Date:**4/21/2020
C:\Users\User\Documents\Munka\Polymer Aerogel\Poliamid aerogel\PA2014\Poroz

Isotherm

Relative Pressure	Volume @ STP [cc/g]	Relative Pressure	Volume @ STP [cc/g]	Relative Pressure	Volume @ STP [cc/g]
1.00790e-02	5.7170	8.59158e-01	44.9512	8.22144e-01	56.1772
4.75990e-02	8.1036	8.82563e-01	47.8721	8.02925e-01	54.2801
7.94070e-02	9.1574	9.02187e-01	50.6330	7.42311e-01	49.8529
1.22248e-01	10.2576	9.20003e-01	53.6295	7.03598e-01	47.2874
1.63564e-01	11.2089	9.38846e-01	60.0415	6.40185e-01	43.2365
2.01040e-01	12.1324	9.59309e-01	65.4803	5.98501e-01	40.7155
2.42522e-01	13.6014	9.67646e-01	68.6960	5.41849e-01	37.3408
3.00476e-01	14.7710	9.80379e-01	75.0061	4.92630e-01	34.0755
3.48928e-01	16.5441	9.81449e-01	79.8054	4.43642e-01	29.5393
4.01242e-01	18.3370	9.85831e-01	83.1997	3.89246e-01	26.5253
4.49477e-01	20.2476	9.83879e-01	84.1286	3.52385e-01	24.4694
4.98675e-01	22.1779	9.79620e-01	84.4282	2.91626e-01	21.3244
5.51502e-01	24.5184	9.70560e-01	79.3501	2.53990e-01	19.3221
6.02369e-01	25.9473	9.60936e-01	77.9543	1.95827e-01	16.1005
6.52832e-01	27.9178	9.43655e-01	72.6227	1.44634e-01	13.1648
6.98435e-01	30.9020	9.20343e-01	67.3702	9.18650e-02	9.8127
7.46953e-01	34.2299	9.01608e-01	63.9543	4.18150e-02	6.1750
7.99223e-01	38.4963	8.81348e-01	61.6146	1.41790e-02	3.1646
8.20033e-01	40.5193	8.61254e-01	59.1982		
8.39440e-01	42.5956	8.41030e-01	57.2814		

Quantachrome NovaWin - Data Acquisition and Reduction
for NOVA instruments
©1994-2010, Quantachrome Instruments
version 11.0

Analysis
Operator: AerogelLab **Date:**2020/05/07
Sample ID: PA(Ca) **Filename:**
Sample Desc: **Comment:**
Sample weight: 0.0524 g **Sample Volume:** 0 cc
Outgas Time: 24.0 hrs **OutgasTemp:** 40.0 C
Analysis gas: Nitrogen **Bath Temp:** 77.3 K
Press. Tolerance: 0.070/0.070 (ads/des) **Equil time:** 300/300 sec (ads/des) **Equil timeout:** 600/600 sec (ads/des)
Analysis Time: 781.3 min **End of run:** 2020/05/07 0:46:59 **Instrument:** Nova Station B
Cell ID: 1 **F/W version:** 0.00

Report
Operator: AerogelLab **Date:**5/7/2020
C:\Users\User\Documents\Munka\Polymer Aerogel\Poliamid aerogel\PA2017CaCl2\

Data Reduction Parameters

Adsorbate Nitrogen **Temperature** 77.350K
Molec. Wt.: 28.013 g **Cross Section:** 16.200 Ĺ **Liquid Density:** 0.808 g/cc

MBET summary

Slope = 13.620
Intercept = 2.359e-01
Correlation coefficient, r = 0.999909
C constant= 58.742

Surface Area = 251.343 m/g

Multi-Point BET Plot

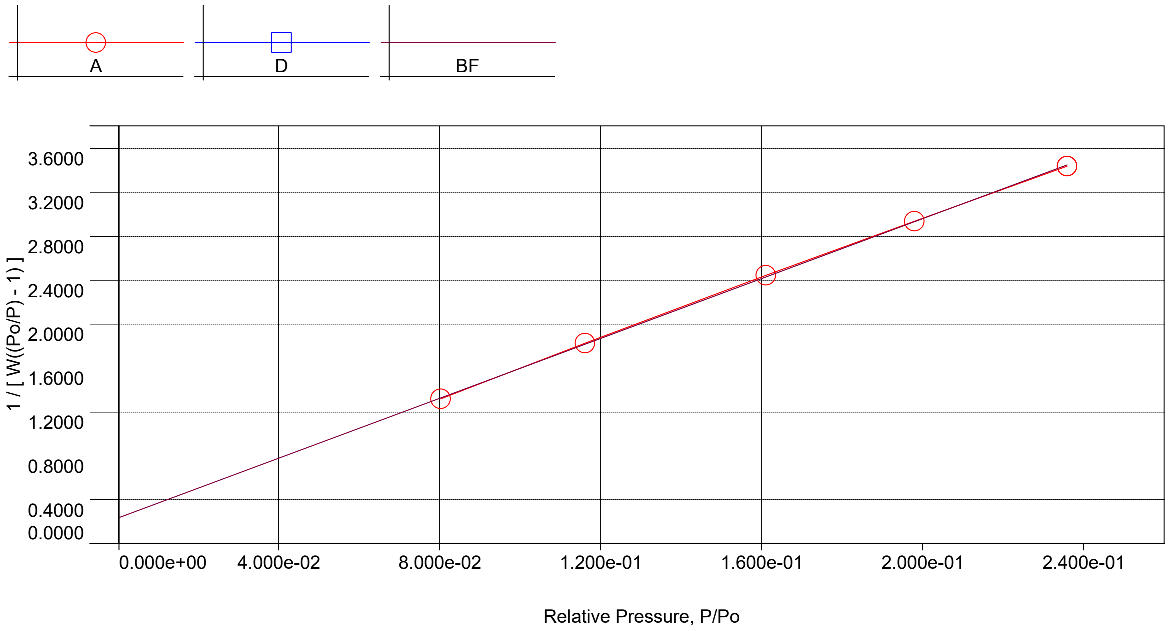

Multi-Point BET

Relative Pressure [P/Po]	Volume @ STP [cc/g]	1 / [W((Po/P) - 1)]
8.03070e-02	53.0534	1.3169e+00
1.16220e-01	57.6200	1.8261e+00
1.60948e-01	62.8672	2.4413e+00

Relative Pressure [P/Po]	Volume @ STP [cc/g]	1 / [W((Po/P) - 1)]
1.97961e-01	67.2863	2.9350e+00
2.35840e-01	71.8435	3.4371e+00

Quantachrome NovaWin - Data Acquisition and Reduction
for NOVA instruments
©1994-2010, Quantachrome Instruments
version 11.0

Quantachrome INSTRUMENTS
Optimizing particle performance

**Analysis**			**Report**		
**Operator:**AerogelLab	**Date:**2019/09/05		**Operator:**AerogelLab	**Date:**4/21/2020	
**Sample ID:** MK_PA2	**Filename:**	C:\Users\User\Documents\Munka\Polymer Aerogel\Poliamid aerogel\PA2014\Poroz			
**Sample Desc:**	**Comment:**				
**Sample weight:** 0.0583 g	**Sample Volume:**	0 cc			
**Outgas Time:** 24.0 hrs	**OutgasTemp:**	40.0 C			
**Analysis gas:** Nitrogen	**Bath Temp:**	77.3 K			
**Press. Tolerance:** 0.070/0.070 (ads/des)	**Equil time:**	300/300 sec (ads/des)	**Equil timeout:**	600/600 sec (ads/des)	
**Analysis Time:** 583.4 min	**End of run:**	2019/09/05 23:51:33	**Instrument:**	Nova Station B	
**Cell ID:** 1			**F/W version:**	0.00	

Data Reduction Parameters

**t-Method**	**Calc. method:** de Boer				
**BJH/DH method**	**Moving pt. avg.:** 3	**Ignoring P-tags below 0.35 P/Po**			
**Adsorbate**	**Nitrogen**	**Temperature**	77.350K		
	**Molec. Wt.:** 28.013 g	**Cross Section:**	16.200 Ĺ	**Liquid Density:**	0.808 g/cc
	**Contact Angle:** 0.0 degrees	**Surf. Tension:**	8.850 erg/cm		

BJH method Desorption dV(log)

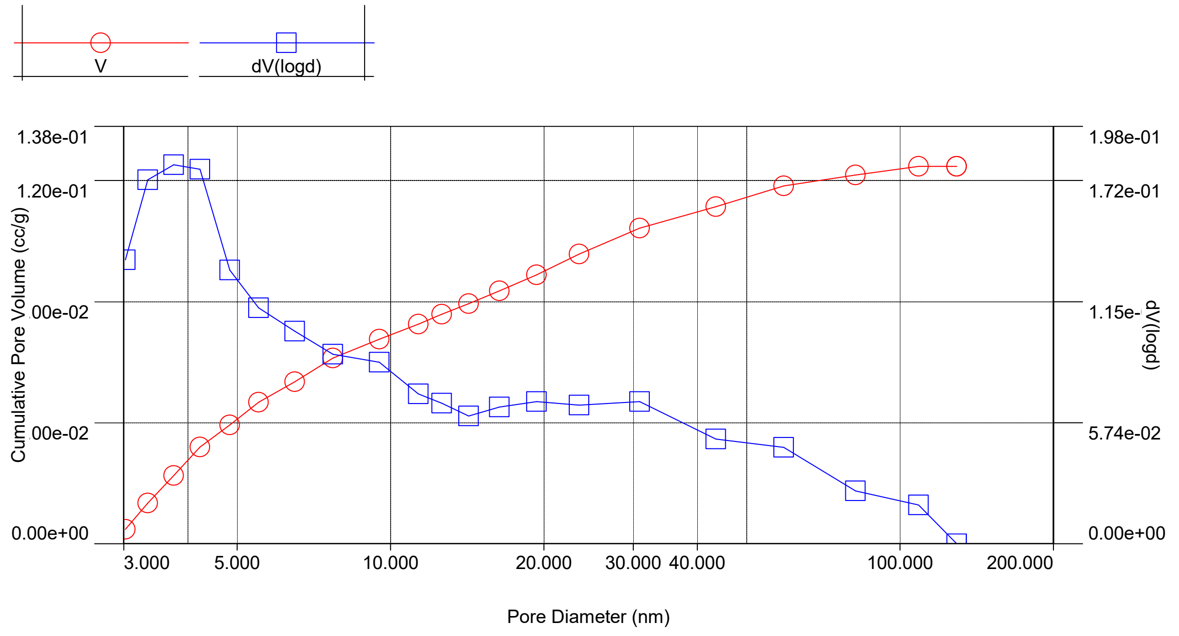

BJH desorption summary

Surface Area = 67.083 m/g
Pore Volume = 0.825 cc/g
Pore Diameter Dv(d) = 3.348 nm

Analysis
Operator: AerogelLab **Date:**2019/09/05
Sample ID: MK_PA2 **Filename:**

Report
Operator: AerogelLab **Date:**4/21/2020
C:\Users\User\Documents\Munka\Polymer Aerogel\Poliamid aerogel\PA2014\Poroz

BJH Pore Size Distribution Desorption

Diameter [nm]	Pore Volume [cc/g]	Pore Surf Area [m‚/g]	dV(d) [cc/nm/g]	dS(d) [m‚/nm/g]	dV(logd) [cc/g]	dS(logd) [cc/g]
3.0236	4.7135e-03	6.2356e+00	1.9326e-02	2.5567e+01	1.3448e-01	1.7790e+02
3.3481	1.3406e-02	1.6073e+01	2.1823e-02	2.5609e+01	1.7261e-01	2.0081e+02
3.7631	2.2527e-02	2.5752e+01	2.0725e-02	2.2143e+01	1.7975e-01	1.9067e+02
4.2277	3.1999e-02	3.4887e+01	1.8799e-02	1.8492e+01	1.7745e-01	1.7293e+02
4.8367	3.9222e-02	4.0987e+01	1.1938e-02	1.0210e+01	1.2988e-01	1.0980e+02
5.5200	4.6733e-02	4.6371e+01	8.8645e-03	6.5505e+00	1.1180e-01	8.1481e+01
6.5033	5.3454e-02	5.0538e+01	6.8649e-03	4.3821e+00	1.0060e-01	6.3100e+01
7.7073	6.1311e-02	5.4602e+01	5.1504e-03	2.7721e+00	8.9814e-02	4.7274e+01
9.5147	6.7502e-02	5.7296e+01	4.0367e-03	1.7833e+00	8.6125e-02	3.7079e+01
11.3437	7.2549e-02	5.9258e+01	2.8642e-03	1.0743e+00	7.0970e-02	2.6301e+01
12.5998	7.5805e-02	6.0292e+01	2.3265e-03	7.5499e-01	6.6581e-02	2.1405e+01
14.2213	7.9320e-02	6.1247e+01	1.8091e-03	5.0311e-01	6.0480e-02	1.6638e+01
16.3874	8.3625e-02	6.2289e+01	1.7360e-03	4.3276e-01	6.4938e-02	1.5959e+01
19.3530	8.8830e-02	6.3346e+01	1.5207e-03	3.2180e-01	6.7514e-02	1.3971e+01
23.4517	9.5671e-02	6.4435e+01	1.1968e-03	2.0651e-01	6.5694e-02	1.0972e+01
30.8988	1.0420e-01	6.5508e+01	9.7891e-04	1.3762e-01	6.7403e-02	8.9559e+00
43.5880	1.1136e-01	6.6267e+01	5.7298e-04	6.3294e-02	4.9595e-02	5.2285e+00
59.2551	1.1817e-01	6.6750e+01	3.5543e-04	2.7197e-02	4.5670e-02	3.2417e+00
81.8039	1.2182e-01	6.6943e+01	1.4787e-04	8.1495e-03	2.5052e-02	1.3496e+00
108.7032	1.2467e-01	6.7083e+01	9.8493e-05	4.8160e-03	1.8357e-02	8.9759e-01
129.2879	1.2467e-01	6.7083e+01	0.0000e+00	0.0000e+00	0.0000e+00	0.0000e+00

Polyamide [Ca(II)] aerogel

Isotherm * Linear

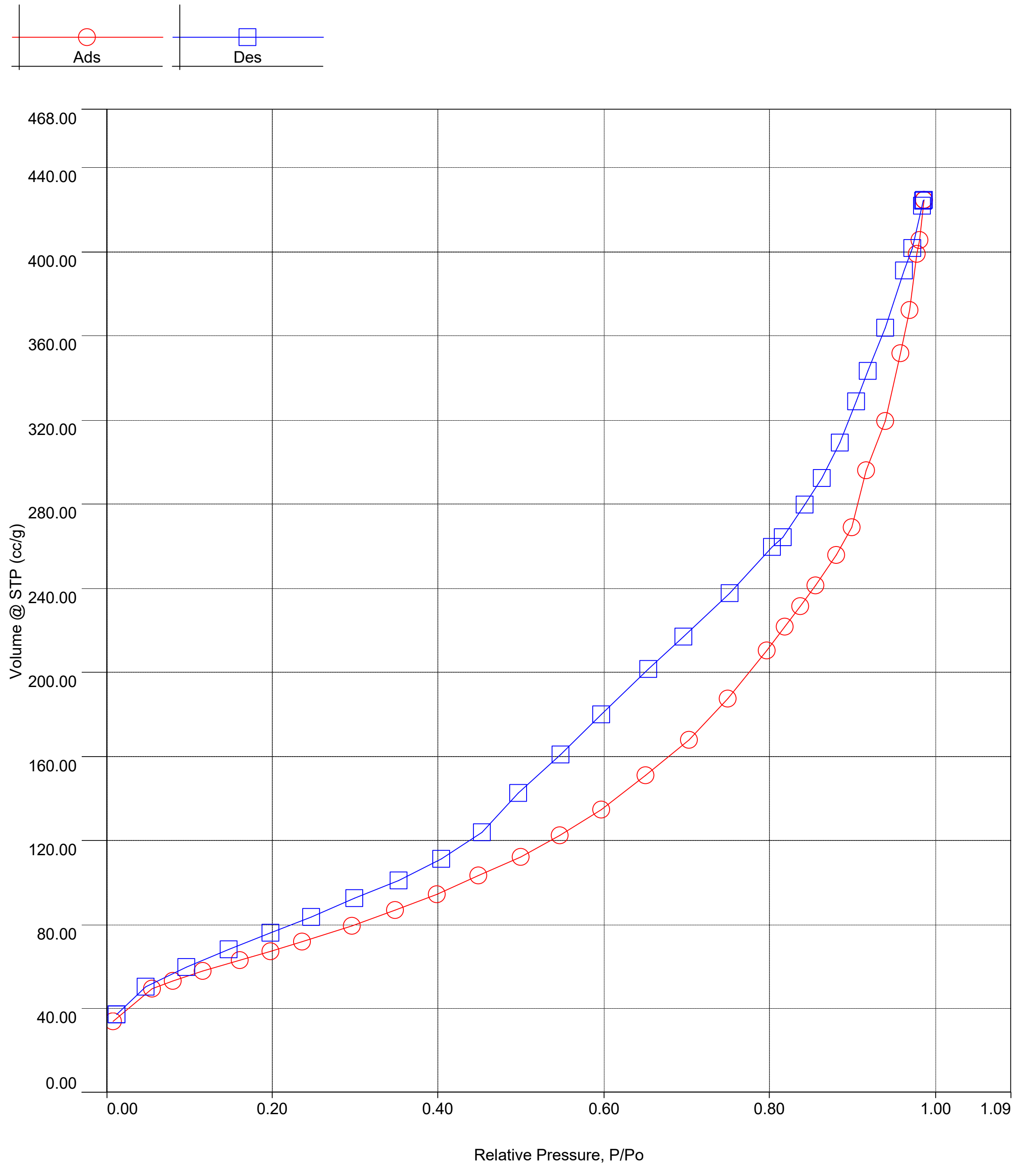

Analysis
Operator: AerogelLab **Date:**2020/05/07
Sample ID: PA(Ca) **Filename:**

Report
Operator: AerogelLab **Date:**5/7/2020
C:\Users\User\Documents\Munka\Polymer Aerogel\Poliamid aerogel\PA2017CaCl2\

Isotherm * Log Scale

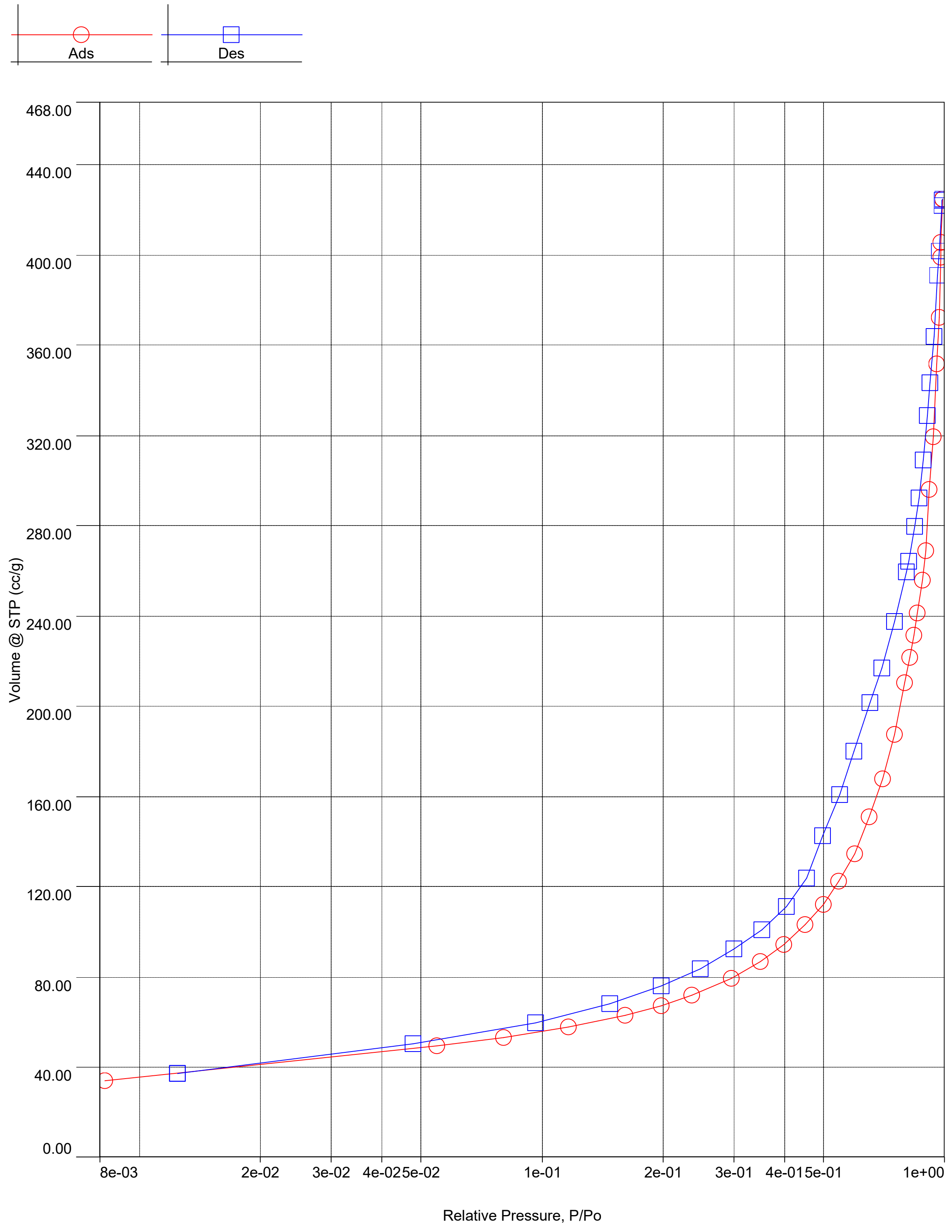

Quantachrome INSTRUMENTS
Optimizing particle performance

Analysis
Operator: AerogelLab **Date:**2020/05/07
Sample ID: PA(Ca) **Filename:**

Report
Operator: AerogelLab **Date:**5/7/2020
C:\Users\User\Documents\Munka\Polymer Aerogel\Poliamid aerogel\PA2017CaCl2\

Isotherm

Relative Pressure	Volume @ STP [cc/g]	Relative Pressure	Volume @ STP [cc/g]	Relative Pressure	Volume @ STP [cc/g]
8.21000e-03	33.7018	8.55519e-01	241.3470	8.15857e-01	264.1803
5.48600e-02	49.1957	8.80121e-01	255.7472	8.03185e-01	259.5487
8.03070e-02	53.0534	8.98653e-01	268.7183	7.51367e-01	237.5732
1.16220e-01	57.6200	9.15918e-01	296.1294	6.96318e-01	216.7372
1.60948e-01	62.8672	9.38679e-01	319.5376	6.53482e-01	201.3149
1.97961e-01	67.2863	9.57277e-01	351.7877	5.96489e-01	179.8053
2.35840e-01	71.8435	9.68631e-01	372.2211	5.48260e-01	160.8738
2.96101e-01	79.5340	9.77446e-01	398.9760	4.96799e-01	142.6006
3.48661e-01	86.8845	9.80190e-01	405.5854	4.53155e-01	123.7865
3.98171e-01	94.4647	9.85642e-01	424.8980	4.04198e-01	111.0529
4.49079e-01	102.9907	9.84303e-01	424.1950	3.52148e-01	101.0774
4.99870e-01	112.2789	9.83946e-01	421.7874	2.99282e-01	92.4259
5.46229e-01	122.1957	9.71208e-01	401.8225	2.47311e-01	83.6567
5.97086e-01	134.7141	9.62088e-01	391.1617	1.98260e-01	76.2546
6.50352e-01	150.8117	9.39393e-01	363.9619	1.47701e-01	68.1058
7.02199e-01	167.9592	9.17949e-01	343.1602	9.63710e-02	59.8336
7.49416e-01	187.5360	9.03923e-01	328.7319	4.78360e-02	50.1758
7.96728e-01	210.2716	8.84540e-01	308.9117	1.24250e-02	37.3136
8.17677e-01	221.7473	8.62740e-01	292.2541		
8.36674e-01	231.3986	8.42221e-01	279.7757		

Quantachrome NovaWin - Data Acquisition and Reduction
for NOVA instruments
©1994-2010, Quantachrome Instruments
version 11.0

Quantachrome INSTRUMENTS
Optimizing particle performance

Analysis
Operator: AerogelLab **Date:** 2019/09/05
Sample ID: MK_PA2 **Filename:**
Sample Desc: **Comment:**
Sample weight: 0.0583 g **Sample Volume:** 0 cc
Outgas Time: 24.0 hrs **OutgasTemp:** 40.0 C
Analysis gas: Nitrogen **Bath Temp:** 77.3 K
Press. Tolerance: 0.070/0.070 (ads/des) **Equil time:** 300/300 sec (ads/des)
Analysis Time: 583.4 min **End of run:** 2019/09/05 23:51:33
Cell ID: 1

Report
Operator: AerogelLab **Date:** 9/24/2020
C:\Users\User\Documents\Munka\Polymer Aerogel\Poliamid aerogel\PA2014\Poroz
Equil timeout: 600/600 sec (ads/des)
Instrument: Nova Station B
F/W version: 0.00

Data Reduction Parameters

Adsorbate **Nitrogen** **Temperature** 77.350K
Molec. Wt.: 28.013 g **Cross Section:** 16.200 Ĺ **Liquid Density:** 0.808 g/cc

MBET summary

Slope = 74.562
Intercept = 1.683e+00
Correlation coefficient, r = 0.999767
C constant= 45.306

Surface Area = 245.675 m /g

Multi-Point BET Plot

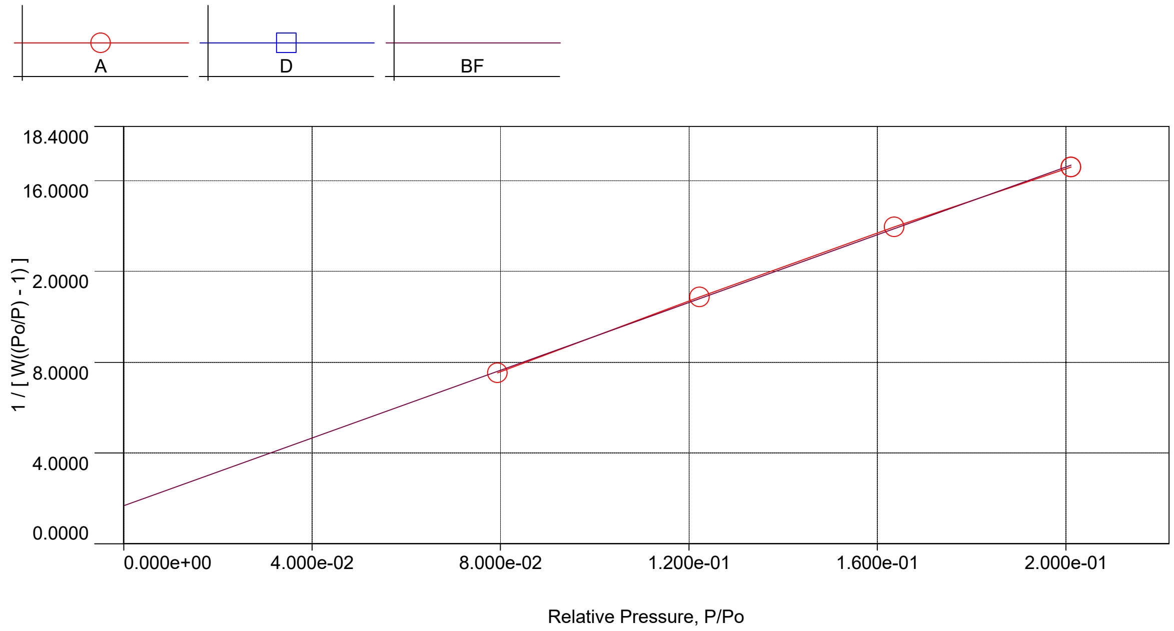

Multi-Point BET

Relative Pressure [P/Po]	Volume @ STP [cc/g]	1 / [W((Po/P) - 1)]	Relative Pressure [P/Po]	Volume @ STP [cc/g]	1 / [W((Po/P) - 1)]
7.94070e-02	9.1574	7.5365e+00	1.63564e-01	11.2089	1.3959e+01
1.22248e-01	10.2576	1.0864e+01	2.01040e-01	12.1324	1.6594e+01

Quantachrome NovaWin - Data Acquisition and Reduction
for NOVA instruments
©1994-2010, Quantachrome Instruments
version 11.0

Quantachrome INSTRUMENTS
Optimizing particle performance

**Analysis**				**Report**		
**Operator:**	AerogelLab	**Date:**	2020/05/07	**Operator:** AerogelLab	**Date:**	5/7/2020
**Sample ID:**	PA(Ca)	**Filename:**		C:\Users\User\Documents\Munka\Polymer Aerogel\Poliamid aerogel\PA2017CaCl2\		
**Sample Desc:**		**Comment:**				
**Sample weight:**	0.0524 g	**Sample Volume:**		0 cc		
**Outgas Time:**	24.0 hrs	**OutgasTemp:**		40.0 C		
**Analysis gas:**	Nitrogen	**Bath Temp:**		77.3 K		
**Press. Tolerance:**	0.070/0.070 (ads/des)	**Equil time:**		300/300 sec (ads/des)	**Equil timeout:**	600/600 sec (ads/des)
**Analysis Time:**	781.3 min	**End of run:**		2020/05/07 0:46:59	**Instrument:**	Nova Station B
**Cell ID:**	1				**F/W version:**	0.00

Data Reduction Parameters

**t-Method**	**Calc. method:** de Boer				
**BJH/DH method**	**Moving pt. avg.:** 3	**Ignoring P-tags below 0.35 P/Po**			
**Adsorbate**	**Nitrogen**	**Temperature**	77.350 K		
	**Molec. Wt.:** 28.013 g	**Cross Section:**	16.200 Å²	**Liquid Density:**	0.808 g/cc
	**Contact Angle:** 0.0 degrees	**Surf. Tension:**	8.850 erg/cm²		

BJH method Desorption dV(log)

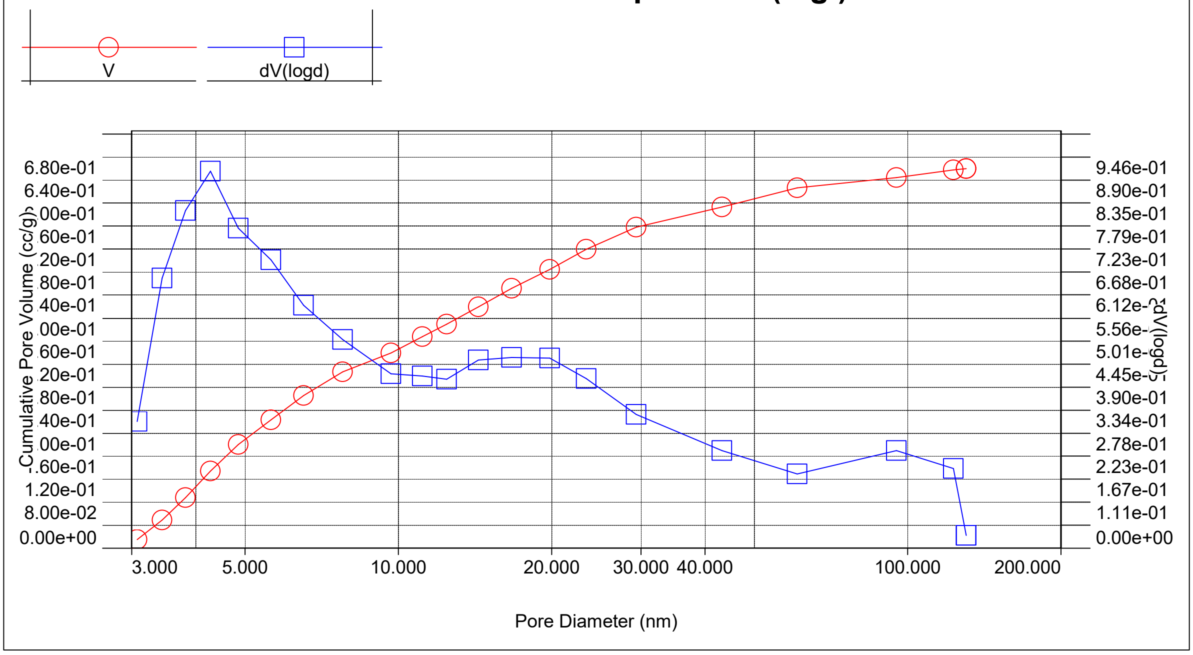

BJH desorption summary

**Surface Area =**	329.520 m²/g
**Pore Volume =**	0.659 cc/g
**Pore Diameter Dv(d) =**	4.284 nm

Quantachrome NovaWin - Data Acquisition and Reduction
for NOVA instruments
©1994-2010, Quantachrome Instruments
version 11.0

Analysis
Operator: AerogelLab **Date:**2020/05/07
Sample ID: PA(Ca) **Filename:**

Report
Operator: AerogelLab **Date:**5/7/2020
C:\Users\User\Documents\Munka\Polymer Aerogel\Poliamid aerogel\PA2017CaCl2\

BJH Pore Size Distribution Desorption

Diameter [nm]	Pore Volume [cc/g]	Pore Surf Area [m‚/g]	dV(d) [cc/nm/g]	dS(d) [m‚/nm/g]	dV(logd) [cc/g]	dS(logd) [cc/g]
3.0755	1.5223e-02	1.9799e+01	4.3388e-02	5.6429e+01	3.0693e-01	3.9918e+02
3.4396	4.8771e-02	5.6926e+01	7.9380e-02	8.9427e+01	6.5286e-01	7.3043e+02
3.8216	8.8163e-02	9.7526e+01	9.1891e-02	9.5803e+01	8.1626e-01	8.4547e+02
4.2838	1.3405e-01	1.4053e+02	9.3681e-02	8.9479e+01	9.1111e-01	8.6187e+02
4.8588	1.8021e-01	1.7828e+02	6.9650e-02	5.8309e+01	7.7393e-01	6.4051e+02
5.6251	2.2314e-01	2.0940e+02	5.5238e-02	4.0812e+01	6.9691e-01	5.0791e+02
6.5342	2.6565e-01	2.3562e+02	3.9851e-02	2.5259e+01	5.8728e-01	3.6620e+02
7.7702	3.0669e-01	2.5632e+02	2.8653e-02	1.5291e+01	5.0485e-01	2.6315e+02
9.6728	3.3941e-01	2.7118e+02	2.0182e-02	9.0691e+00	4.2117e-01	1.8530e+02
11.1520	3.6799e-01	2.8186e+02	1.6486e-02	6.0710e+00	4.1630e-01	1.5145e+02
12.4497	3.8980e-01	2.8863e+02	1.4030e-02	4.4787e+00	4.0813e-01	1.2905e+02
14.3490	4.1959e-01	2.9695e+02	1.3888e-02	3.9574e+00	4.5463e-01	1.2768e+02
16.7004	4.5171e-01	3.0448e+02	1.1932e-02	2.8857e+00	4.6134e-01	1.0966e+02
19.8302	4.8477e-01	3.1128e+02	1.0263e-02	2.1404e+00	4.6018e-01	9.4311e+01
23.3341	5.1968e-01	3.1721e+02	7.8291e-03	1.4012e+00	4.1023e-01	7.1878e+01
29.3217	5.5774e-01	3.2202e+02	4.9645e-03	7.3200e-01	3.2349e-01	4.5453e+01
43.1226	5.9273e-01	3.2566e+02	2.7337e-03	3.0949e-01	2.3631e-01	2.4932e+01
60.7407	6.2599e-01	3.2802e+02	1.4288e-03	1.1167e-01	1.7952e-01	1.2960e+01
95.1971	6.4450e-01	3.2893e+02	1.0763e-03	4.9658e-02	2.3539e-01	9.8416e+00
122.9646	6.5728e-01	3.2945e+02	7.3027e-04	2.5686e-02	1.9269e-01	6.6750e+00
130.0026	6.5937e-01	3.2952e+02	1.0137e-04	3.1189e-03	3.0324e-02	9.3303e-01
